# Improving goal striving and resilience in older adults through a personalized metacognitive self-help intervention: a protocol paper

**DOI:** 10.1186/s40359-023-01259-3

**Published:** 2023-08-04

**Authors:** Lotte P. Brinkhof, K. Richard Ridderinkhof, Jaap M. J. Murre, Harm J. Krugers, Sanne de Wit

**Affiliations:** 1 Department of Psychology, Faculty of Behavioral and Social Sciences, University of Amsterdam, Amsterdam, Netherlands; 2 Centre for Urban Mental Health, University of Amsterdam, Amsterdam, Netherlands; 3 Amsterdam Brain & Cognition (ABC), University of Amsterdam, Amsterdam, Netherlands; 4 Faculty of Science, Swammerdam Institute for Life Sciences, University of Amsterdam, Amsterdam, Netherlands

**Keywords:** Behavior change, Quality of life, Mental well-being, Self-help intervention, Older adults, Habit, Implementation intention, Successful aging

## Abstract

**Background:**

Successful aging is often linked to individual’s ability to demonstrate resilience: the maintenance or quick recovery of functional ability, well-being, and quality of life despite losses or adversity. A crucial element of resilience is behavioral adaptability, which refers to the adaptive changes in behavior in accordance with internal or external demands. Age-related degradation of executive functions can, however, lead to volition problems that compromise flexible adjustment of behavior. In contrast, the reliance on habitual control has been shown to remain relatively intact in later life and may therefore provide an expedient route to goal attainment among older adults. In the current study, we examine whether a metacognitive self-help intervention (MCSI), aimed at facilitating goal striving through the gradual automatization of efficient routines, could effectively support behavioral adaptability in favor of resilience among older adults with and without (sub-clinical) mental health problems.

**Methods:**

This metacognitive strategy draws on principles from health and social psychology, as well as clinical psychology, and incorporates elements of established behavioral change and activation techniques from both fields. Additionally, the intervention will be tailored to personal needs and challenges, recognizing the significant diversity that exist among aging individuals.

**Discussion:**

Despite some challenges that may limit the generalizability of the results, our MCSI program offers a promising means to empower older adults with tools and strategies to take control of their goals and challenges. This can promote autonomy and independent functioning, and thereby contribute to adaptability and resilience in later life.

**Trial registration:**

Pre-registered, partly retrospectively. This study was pre-registered before the major part of the data was collected, created, and realized. Only a small part of the data of some participants (comprising the baseline and other pre-intervention measures), and the full dataset of the first few participants, was collected prior to registration, but it was not accessed yet. See: https://osf.io/5b9xz

**Supplementary Information:**

The online version contains supplementary material available at 10.1186/s40359-023-01259-3.

## Background

### Resilience and behavioral adaptability as critical element

Older adults’ mental well-being and quality of life (QoL) may be jeopardized by various age-related challenges and transitions (e.g., loss of spouse or functional abilities) if these are not adequately managed and controlled [1, 2]. Accordingly, successful aging is often linked to an individual’s ability to demonstrate resilience: successful adaptation in the face of challenges [3–7]. Resilience can be conceptualized as the maintenance and/or quick recovery of functional ability, (mental) well-being and QoL despite losses or adversity [1, 2, 8, 9], and has been considered a defense mechanism against mental health problems (but also [10–12] for their perspectives on resilience).

Although much emphasis is generally put on the importance of *psychological adaptability* (e.g., exhibiting adaptive coping styles and self-management abilities, or having an optimistic and positive frame of mind; [9]), *behavioral adaptability* also constitutes a critical element of resilience. It refers to the adaptive changes in behavior in accordance with internal or external demands [13, 14]. In later life, the (impending) loss of a spouse or good friend may, for instance, require adjustments in daily life behaviors to increase opportunities for other social contacts to diminish or prevent feelings of loneliness. Similarly, to delay physical decline or to alleviate depressive symptoms, older adults may scale up the number of hours spent on (physical) activities [15–18]. Age-related degradation of executive functions can, however, lead to volition problems that compromise flexible, goal-directed adjustment of behavior (e.g., failing to get started; [19–24]). These may be exacerbated by mental health problems, including depression or apathy, which is defined as a quantitative reduction in goal-directed, non-routine behaviors due to as loss of effort/initiative, interest and/or emotional reactivity [25–27]. In contrast, however, the reliance on efficient habitual control has been shown to remain relatively intact in later life [20, 28], and may therefore provide an expedient route to goal attainment among older adults [29].

In the current study, we examine whether a metacognitive self-help intervention (MCSI), aimed at facilitating goal striving through the gradual automatization of efficient routines, could effectively support behavioral adaptability in favor of resilience among older adults with and without (sub-clinical) mental health problems. This MCSI is based on insights from health & social psychology and clinical psychology and adopts an integrated approach by combining components of existing behavioral change/activation interventions from both fields. The idea is that individuals learn a strategy that can help them to set and strive for self-identified goals. To accommodate for the large individual differences that exist among aging individuals, a personalized framework is used to tailor the intervention to personal needs and challenges.

### Improving behavioral adaptability through habit formation

When a specific action is consistently performed in response to a situational cue, an associative link between the situation and that action (i.e., stimulus–response link) is formed [30]. This process is known as habit formation and enables individuals to automate behaviors without the need for conscious planning. Habit formation as a potential mechanism to foster behavioral adaptability has received widespread attention, particularly for samples with volition problems [31]. A particularly prevalent volition problem among older individuals pertains to action initiation [32]. It has been hypothesized that this is due to the stability of older adults’ lives and the regularity with which they encounter situational cues, making it harder to initiate an intended change [32, 33]. For instance, increasing exercise behavior in daily life likely requires one to adjust some deep-seated routines (e.g., taking the stairs, rather than the elevator; after lunch going for a walk first, instead of putting on the TV immediately). Therefore, it may be particularly challenging for older adults to initiate new target behaviors that compete with existing habits. On the other hand, once the target behavior has been initiated, the stability of older adults’ lives may help to automatize and maintain this new behavior [32].

A useful strategy to overcome difficulties with action initiation and facilitate automatized goal striving is by forming so-called ‘implementation intentions’ (IIs; [21]): if–then plans that specify a behavior to be performed in response to an anticipated cue *(‘If situation Y arises, then I will initiate behavior X’*; [34, 35]). Such plans are thought to operate by heightening the cognitive accessibility of a situation cue (or opportunity to act) and by forging a mental association with a desired behavior, such that this is automatically elicited when the situation is subsequently encountered [34–37]. For instance, after formulating the following plan: “If I enter my building, then I will take the stairs to my floor”, entering the building becomes a trigger for walking up the stairs, and one does not have to deliberate about when or how to act. This increases the likelihood of consistent repetition [21, 38], and thereby facilitates habit formation [39, 40].

IIs have been widely applied in health psychology, and have been shown to facilitate goal attainment among the general population, as well as specific subgroups [21]. Interestingly, IIs are considered to be particularly helpful for individuals whose self-regulatory skills are compromised [21], thereby serving as a compensatory strategy for those in strongest need of assistance. This has been supported by a number of studies showing that IIs can help to overcome ego-depletion [41], and promote goal attainment among those suffering brain damage or drug addiction [42, 43], as well as improve prospective memory performance among those with low executive functioning [44, 45] or fluid mechanics (i.e., those cognitive functions that tend to decline with age; [46]). Accordingly, IIs have been suggested as a means to compensate for age-related decline in prospective memory [47]. Indeed, several studies have already provided promising results among older adults [48–51], showing that IIs can foster new sets of actions in favor of resilience in later life (e.g., improve physical activity; [51]).

### Using implementation intentions to support mental health

Another group that may particularly benefit from IIs and automatization of adaptive behaviors are those with underlying mental health problems. Previous research has emphasized how mental health problems may exacerbate goal striving challenges (e.g., see [27, 52, 53]). This may be especially the case in many aging individuals, who already experience a natural degradation of their (goal-directed) self-regulatory processes. This is a critical issue, as goal striving and adaptive behavior change (e.g., engaging in social/physical activities) can effectively break or even reverse the downward spiral to mental health problems that is most prevalent among older adults (e.g., apathy, depression, loneliness; [16, 17, 52, 54–59]).

In clinical practice, promoting adaptive routines is part of *behavioral activation treatment*, which is built on the premise that engaging in behaviors that connect people to sources of positive reinforcement can improve mental health (e.g., alleviating depressive symptoms, increasing social connectedness; [16, 17, 58]; based on Lewinsohn’s theory of depression [60]). Specifically, behavioral activation encourages individuals to engage in pleasurable, mood-independent, pre-planned activities, and therefore overlaps largely with our primary goal of using IIs to support behavioral adaptability in favor of resilience. Behavioral activation is often incorporated in cognitive behavioral treatment (e.g., Beck’s Cognitive Therapy; [61]), and may be an important driving force behind its efficacy [62, 63].

A critical element of behavioral activation treatment is monitoring of daily activities and mood, followed by identifying adaptive behaviors that could restore an adequate schedule of positive reinforcement (e.g., calling daughter; going for a walk every day; see [16] for manual). Such a personalized (reward-driven) framework may also provide a useful tool for improving the efficiency of IIs, especially when applied more broadly, in a metacognitive way. Reversely, IIs have also been suggested to boost behavioral activation by stimulating the actual execution of the personally identified activities [53]. That is, while behavioral activation treatment encourages to include the identified activities in their daily schedule (e.g., ‘at 9 a.m. on Monday’), they are not instructed to link this to a specific situation, pre-existing routine or other consistent opportunity to act (e.g., ‘If I have finished my breakfast’; [53, 64]; and also see [16]), which may result in lower than desired enactment. Hence, by incorporating these behavioral activation principles (i.e., monitoring of daily activities and mood, and encouragement of engagement in rewarding, personally relevant activities that match with intrinsic values) into an II intervention, the formation of persistent habits may be accelerated and behavioral adaptability in favor of resilience, and consequently better mental well-being, QoL and mental health, may be more effectively supported in the older population.

Importantly, IIs have been found to be effective among clinical populations. In previous studies, IIs were either targeted at reducing behaviors that were part of the symptomatology (e.g., [65, 66]) or, most commonly, focusing on improving adaptive behaviors that could accelerate recovery and treatment of symptoms (e.g., psychotherapy attendance, increasing social/physical activities, relaxation under stressful circumstances; [53, 67–70], as well as the prevent relapse (e.g., [71, 72]). A meta-analysis of Toli and colleagues [27] demonstrated that IIs effectively support goal attainment among clinical samples, with the effect size being larger than has been found for non-clinical populations [21]. Interestingly, IIs have been suggested to be particularly beneficial for mental disorders that are characterized by low levels of executive functioning [63], lending support for the idea that older adults with (sub-clinical) mental health problems may also be a subgroup that can largely benefit from IIs. Yet, how mental health problems at an advanced age influence the effectiveness of IIs has not been established.

### Strategic planning of adaptive behaviors

The formulation of a personalized II involves multiple steps. *Firstly*, as IIs are only effective when underpinned by strong intentions to change behavior [34–36], one should define a clear goal intention (GI; e.g., ‘My *goal* is to engage in more social/physical activities’) that aligns with the motivation to do so (‘I *want* to engage in more social/physical activities’). Importantly, this GI should be somewhat challenging, as forming IIs does not provide additional benefit when the goal is relatively easy to achieve [73], or is performed frequently already [74]. *Secondly*, one should decide what goal-directed behavior would be appropriate to achieve the goal. This behavior should be realistic, concrete and not overly complicated (e.g., ‘walking for at least 15 min on a daily basis’, or ‘going to a local community center’). This overlaps with the SMART criteria for goal setting in the context of cognitive behavioral therapy (specific, measurable, achievable, relevant and time-bound; [75]). *Thirdly*, this action should be linked to a specific cue that provides a good opportunity to act on the behavior. Selecting an appropriate cue for the if-portion entails deciding which of the many possible (consistent) opportunities is most useful and effective to achieve one’s goal. It has been recommended to select an event-based cue (i.e., situational), rather than a time-based cue (e.g.,’after breakfast’ instead of’at 9 am’) as these are more salient and do not involve active monitoring of the time of day and thus reduce the likelihood of missing the critical situation, especially among older adults [76]. The likelihood of cue encounter and plan enactment may also be enhanced by choosing a cue that takes place on a daily, rather than weekly, basis (e.g., after breakfast, after lunch, after dinner; [51]).

### Metacognitive use of implementation intentions in the older population

Typically, in previous studies, participants were guided through the II formulation process, after which they were encouraged to work with a specific plan for a number of weeks to test its effectiveness. In this way, one may learn how to form an II to strive for one specific goal. However, to promote resilience and independent functioning in older age, it is critical that older adults can use this self-regulation strategy independently, as a *metacognitive (self-help) intervention (MCSI),* to set and strive for any self-identified goal and thereby tackle a multitude of challenges in different domains [77–79]. This necessitates a multi-pronged approach, in which individuals are encouraged to *monitor* opportunities for behavioral change in their daily life, and supported in forming effective IIs. In addition, the wide application of IIs to everyday life behaviors likely requires a certain extent of *plan evaluation* in terms of its effectiveness. That is, while a plan may be effective at the start, circumstances or personal needs may change over time, such that it could lose its feasibility and effectivity. For instance, the situational cue may no longer provide a good opportunity to act (e.g., not very consistent, easily missed). Alternatively, the goal-directed behavior may become excessively costly or impossible to carry out, or no longer align with one’s intention, such that encountering the situational cue will probably not elicit the desired response [36]. The concreteness and consequently inflexibility that is inherently linked to implementation intentions may then even provide a disadvantage, rendering individuals less inclined to adjust their behavior when the situation calls for this [80, 81]. By encouraging individuals to evaluate their progress, reflect upon their plan to determine whether it is still relevant, and formulate a new, more suitable plan when necessary, both the effectiveness and wide applicability of IIs can be enhanced [78, 82]. Importantly, it is expected to increase the volitional nature of their plans and the experienced autonomy [74, 83].

To prompt and support individuals to use *monitoring*, *planning* and *plan evaluation* principles both during and after the intervention period, a comprehensive and logically structured manual is provided as part of the MCSI. This manual emphasizes the importance of being able to adequately adjust one’s behavior to internal and external demands in later life, and includes a detailed description on how IIs can provide an easy tool to accomplish this. In addition, it explains how monitoring of new opportunities for behavioral change is integrated in the intervention (i.e., by answering daily questions about one’s satisfaction with several lifestyle domains, mood and experienced daily events), and describes the most relevant questions one can ask oneself to carefully evaluate the effectiveness and usefulness of their II.

### Current scope: early intervention for non-clinical and sub-clinical samples

The potential of using II as a key element of a MCSI has been suggested in previous literature [78, 84, 85]. Yet, it remains to be established whether it supports behavioral adaptability (as critical element of resilience) among older adults. As this intervention is intended to support self-management of daily life behaviors that support functional ability, well-being and QoL, it may provide a promising tool for the prevention or alleviation of emerging mental health problems. Early treatment of symptoms of depression or loneliness can potentially prevent their escalation [55, 86–88]. Importantly, it may also reduce the need for intensive therapy among clinical samples, which usually involves professional clinical supervision. In this way, an effective MCSI may help to alleviate pressure on existing systems of care ([89]; also see [90]) and provide an efficient route towards better public health.

In the current study, we focus on non-clinical and sub-clinical older adults, who do not (yet) require help of a trained clinician, to examine the potential of the metacognitive (IIs) self-help intervention as an early intervention.

### Current study: aims

**Fig. 1:**
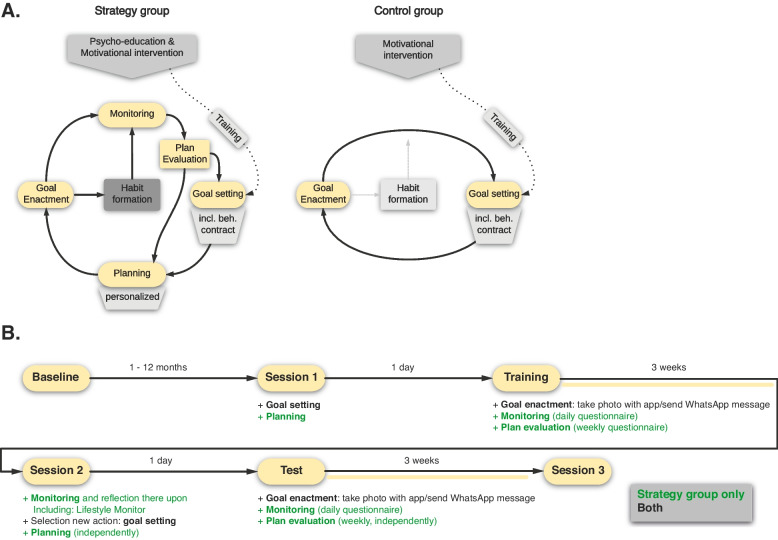
Experimental design. The key elements of the study are shown in the top diagrams, with the experimental metacognitive strategy and control group in the left and right panel, respectively. When these key elements are included within the program, is shown in the bottom section

In the current study, we train older adults to use our MCSI to facilitate striving of a pre-determined goal (i.e., walking for at least 15 min on a daily basis; training phase), after which they are prompted to deploy the same strategy for another (personal) everyday life challenge (test phase). In this way, we intend to coach older adults in how to manage current, as well as future demands and challenges that may cross their path by tailoring this strategy to their personal goals and obstacles. The intervention combines several behavior change principles, from social & health psychology [35] and clinical practice, focusing on some of the techniques that are described in the behavioral activation treatment for depression manual of Lejuez and colleagues [16] (also see [58] for modification example). We strive for a short, but comprehensive and effective intervention with IIs as the central ingredient, inspired by elements of behavioral activation. In some cases, these elements have a more general character, since we do not focus on depression specifically, but on mental well-being, QoL and alleviation of mental health problems in general. The effectiveness of the intervention will be assessed by comparing an experimental metacognitive strategy group with a group that solely formulates a goal intention to support goal enactment (i.e., the control group; see Fig. 1).

The central aim of this study is to test the effectiveness of the MCSI and determine whether it can indeed effectively support goal striving in favor of resilience among older adults. To this end, we examine whether the MCSI can effectively support behavioral adaptability (aim 1a) and whether mental well-being, QoL are thereby improved, and mental health problems reduced (aim 1b). Thus, we evaluate one’s level of resilience by looking at several outcome variables, referred to as outcome based resilience [2, 91, 92]. We assume that those who are more equipped to adjust their behavior in accordance to personal goals and challenges, are more resilient, and thus report more favorable levels of these outcome variables. *Aim 1a* will be established by evaluating changes in both *phase-dependent* (e.g., training or test) and *phase-independent* variables. Phase dependent variables include the frequency of the target behavior, temporal regularity of the performance and the perceived automaticity, whereas phase independent variables comprise self-efficacy (i.e., the belief that one can successfully execute the behaviors required to produce an outcome; [93]), self-management ability, the tendency to engage in if–then planning and lifestyle satisfaction [94, 95]. We hypothesize that individuals in the strategy group will show a higher (and more consistent) frequency of behavior, as well as better improvement in perceived automaticity during both the training and test phase. Moreover, self-efficacy, self-management ability, the tendency to engage in if–then planning and lifestyle satisfaction are expected to show a larger improvement for this group.

*Aim 1b* will be determined by examining the direct impact of the intervention on mental well-being and QoL, and several outcome variables that tap onto different kinds of mental health problems, including depressive symptomatology, loneliness, and apathy. We expect that the strategy group will show more beneficial effects of the intervention (after the test phase) than the control group, as reflected in a larger reduction in depressive symptomatology, loneliness, and apathy, and a greater increase in mental well-being and QoL. In addition, we will also quantify the extent to which the *behavioral adaptability variables* (except for regularity) act as intermediate variables and therefore explain the potential change in mental well-being, QoL and mental health outcomes. These phase-dependent behavioral adaptability variables either reflect competencies or inclinations that are associated with higher self-sustainability and adaptability (self-efficacy, self-management ability, and if–then planning) or imply success experiences (lifestyle satisfaction, performance of behavior and perceived automaticity). We hypothesize that frequency, perceived automaticity, and all *phase- independent variables* mediate the effects on the health outcome variables. Altogether, this allows us to identify the mechanisms that putatively underlie the intervention effects on mental well-being, QoL and mental health problems, and it can help us to determine ways to further improve the intervention, especially when mediating variables are not affected.

In addition, we will examine whether mental health problems (assessed prior to the intervention) moderate the effectiveness of the MCSI (Aim 2). A previous meta-analysis suggest that the effects of IIs are larger for those with underlying mental health problems than for non-clinical samples [21, 27]. We will for the first time *directly* assess the modulating effect of underlying mental health problems on the effectiveness of IIs, in combination with other behavior change components. Because of the scarcity of previous research, we will assess the role of underlying mental health problems in an exploratory fashion, without strong a priori hypotheses.

We also aim to shed light on the extent to which a certain level of cognitive functioning may be necessary for the effectiveness of this MCSI (Aim 3). Indeed, as elaborated previously, IIs are generally considered to be particularly helpful for individuals whose self-regulatory skills are compromised [21]. Nonetheless, a certain level of cognitive resources may also be necessary to effectively deploy IIs, especially when applied in a metacognitive way. Evidence for this comes from a study of Burkard and colleagues [96], who showed that IIs were only efficient among older individuals with relatively high working memory capacity. This could explain why some studies have found beneficial II effects for the young-old, but not the old-old (e.g., [97]). This suggests that when cognitive resources are extensively compromised, this may form a boundary condition for the effectiveness of the metacognitive use of IIs. In the current study, we will address this matter by evaluating the relation between working memory capacity and the effectiveness of the MCSI, where we test two competing hypotheses: (1) that the MCSI will be more effective at facilitating behavioral adaptability in favor of resilience (as reflected in better QoL, mental well-being/health) for those with high working memory capacity, in line with the position that (some level of) working memory is essential for such a self-help strategy to be effective, and (2) that this MCSI will be more effective for those low in working memory capacity, in line with the contention that such a self-help strategy can serve as a compensatory strategy for those with low.

### Additional aims

By teaching individuals how to effectively use the MCSI to manage demands and challenges that cross their path (i.e., supporting behavioral adaptability as critical element of resilience), they may also develop a general buffer against stressful events or perturbations, thereby fostering resilience to daily stressors (i.e., tapping onto the psychological adatability element of resilience). Firstly, once healthy and efficient routines are formed, these behaviors will be relatively insensitive to stress [29, 98], thereby maintaining the provisions for good mental well-being/health in the face of adversity, even when self-control processes might be compromised. Secondly, if individuals feel more in control of their behavior and experience elevated levels of self-efficacy, they may more easily adapt to (or even prevent) such events, also promoting good mental well-being/health. To this end, we will also examine whether mastering the MCSI may alleviate the effects that daily stressors/hassles on psychological distress and daily mood. Daily stressors refer to experiences and conditions of daily living that are appraised as salient, harmful or threatening to an individual’s well-being” [99]. One’s level of psychological distress refers to a state of emotional and psychological discomfort or disturbance that is characterized by non-specific symptoms of anxiety and depression, which can persist for a longer period of time; daily mood pertains to the transient emotional states or feelings that individuals experience on a day-to-day basis.. We hypothesize that the MCSI will indeed alleviate the effects that stressors/hassles can have on individuals’ psychological distress and daily mood. To better interpret potential changes in one’s ability to deal with such stressors/hassles, we will also consider the impact of one’s baseline resilience level (i.e., how well one was dealing with stressors prior to the intervention) and scores on two psychological appraisal style constructs. When encountering a stressor, several thoughts and thinking processes can occur [92, 100], and these likely have a great impact on how well individuals may be able to show resilience against daily stressors/hassles. We expect that more positive thinking processes (PASSp, process focused) and thoughts (PASSc, content focused) are associated with better stressor-coping.

## Methods/Design

### Sample characteristics

Participants are recruited in diverse ways, resulting in three types of participants.

- **Type A.** All participants of 65 years or older that completed the main inventory of a larger project on successful aging and resilience [101], and indicated that their personal data could be stored to be invited for follow-up studies, were invited to fill out an additional set of questionnaires several months later (i.e., follow-up study). A number of these questions also comprise some crucial (baseline) measures of the current study. All participants that completed the follow-up study are invited to participate in the current study and are considered ‘type A’ participants if they enrolled in the current study within 12 months after completing the follow-up survey.
- **Type B**. All participants of 65 years or older that are not yet part of the overarching projects’ participants pool, but are interested to participate in both the main inventory and the current, can also participate. If so, participants are instructed to enroll in the overarching project by completing the main inventory, after which they can express their interest in participating in the current study as well. All participants that enroll in the current study within 30 days after completing the main inventory will be ‘type B’ participants.
- **Type C.** In case participants do not enroll in the current within 30 days after completing the main inventory, and/or completed the follow-up questionnaire more than 12 months ago, participants are considered ‘type C’ participants.

In all cases, we indicate to participants that we are looking for people who are motivated to learn a new strategy to change their behavior in accordance with personal goals. To rule out a possible influence of completing the baseline measurements at varying times and the presence of different other questions alongside those used in the current study for participants categorized as type A and B, we will conduct an exploratory ANOVA and statistically compare scores on all baseline questionnaires and scales. Initially, having an Android mobile phone was an important inclusion criterion, since participants have to download a mobile application that is only available at Android devices (see our pre-registration). After submitting this pre-registration, and collecting data of some of the participants, we found a solution for iPhone users (see Methods for more details). From that point onwards, iPhone users that have previously indicated to be interested, are also invited to participate. All participants need to provide informed consent for the collection and use of their data during the current study, as well as the use of data provided in previous parts of the overarching study (i.e., main inventory and follow-up study for type A participants).

### Sample size

G*Power was used to perform an priori power analyses to establish the sample size needed to obtain a power (1 – β) of 0.8, with *α* = 0.05 and effect size *f* = 0.25 (medium) for a mixed ANOVA, the statistical test used to analyze the patterns of change in behavioral frequency and perceived automaticity (part of aim 1a). The minimum required sample size (of all analyses) was *N* = 98, suggesting a minimum of *n* = 49 is needed per intervention group. Therefore, our target sample is 100 participants, which represents a feasible number of participants to recruit and test (note that much more participants would be unfeasible considering several practical issues, e.g., costs and investment). Assuming that not all will complete the entire intervention, we expect we need to recruit a slightly higher number of participants – up until we reach a total of 100 (50 strategy group, 50 control group – stopping rule).

Importantly, some of the questions we aim to answer require path-analyses approaches. Such statistical procedures generally require much larger sample sizes, limiting our possibilities of obtaining enough statistical power to evaluate very complex models. For that reason, we will use a two-step approach for our complex analyses. In the first step, only two mediators will be included, of which the results will be interpreted in a *confirmative fashion*. The other mediators of interest will be added in a second step. Results of this complete and more complex model will be interpreted in *an exploratory fashion*. This allows us to infer null hypotheses for negative results for the variables included in the first step, but also to test other mechanism that may explain intervention effects that we believe can still have scientific merit, even though our model may not meet conventional requirements for statistical power (see Analyses for more details on the specific variables). A Monte Carlo power analysis for indirect effects revealed that a *two* parallel mediation model, with *N* = 100 and underlying relationships of *r* = 0.40 (except X ~ Y, *r* = 0.20), should result in a power of approximately 0.81 to 0.83.^1^

### Procedure

**Fig. 2:**
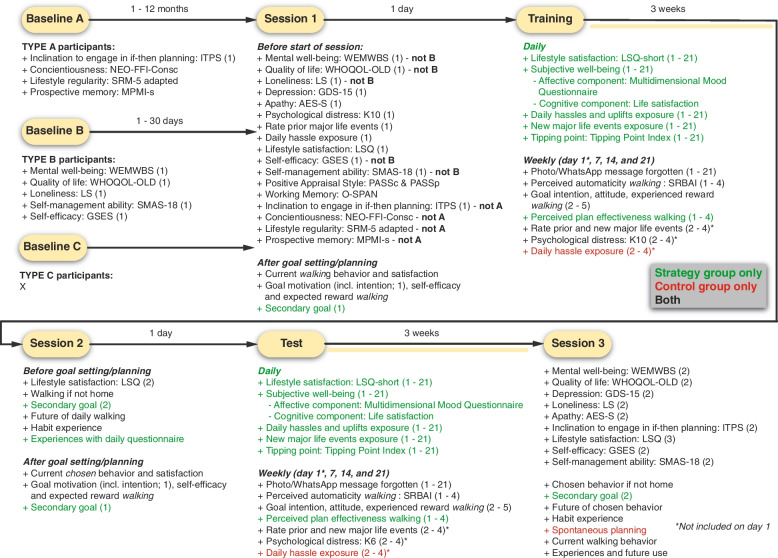
Overview of questionnaires prior and during sessions/phases of the study. Numbers in parentheses correspond to their respective assessment instance, starting from (1) for the first time, (2) to the second time, etc. Details on the materials can be found in Supplement A. Some important concepts that have not been elaborated on in the main text are, however, clarified here. Photo/WhatsApp message forgotten: in case participants forget to take a picture/send a WhatsApp message before/while performing their target behavior, they can provide the date(s) manually at the end of each week, such that the frequency measure can be adapted. To ensure that we will observe participants’ natural adherence without the influence of daily reminders, we refrain from asking participants whether they have taken a photo/send a WhatsApp message on a daily basis. Habit experience refers to the extent to which the act of walking/performing the chosen behavior on a daily basis is considered to be a habit by the participants, as indicated on a scale ranging from ‘not at all’ to ‘very strongly’. Secondary goal refers to a subordinate goal that individuals may have when their goal/plan entails inhibiting an old habit by replacing it with something positive/good. For instance, a person may want to reduce her snacking behavior (secondary goal) by eating an apple after dinner (primary goal), rather than crisps

The intervention consists of multiple elements (Fig. 1.), divided over several sessions (Fig. 2.). Each of these sessions is build-up differently and includes several questionnaires that have to be completed by the participants. Importantly, all sessions are individual, allowing for the personalization of the intervention.

#### Baseline

Weeks to months prior to session 1, type A participants’ inclination to engage in if–then planning was assessed. In addition, some personality trait-like constructs were examined, including conscientiousness, lifestyle regularity, prospective memory ability, as well as the use of internal and external PM strategies to remember intentions. Days to weeks prior to session, type B participants’ mental well-being, QoL, loneliness, self-management ability and self-efficacy is assessed. Hence, these measures are not included in the questionnaires that have to be completed prior to session 1 for this subgroup of participants specifically. Finally, for type C participants, no previous data is used and considered as baseline measure, and all relevant measures are therefore added to the pre-session 1 questionnaire.

#### Assignment to groups

Participants are equally divided (in alternating order based on registration) over two groups: a metacognitive strategy group and an active control group that only formulated a goal intention. Individuals assigned to the strategy group receive a manual, comprising all relevant information and steps of the intervention, several days before session 1.

#### Session 1 (1.5 – 2 h)

The first session takes place online (via Microsoft Teams). All participants (A, B, C) are instructed to complete a list of questionnaires prior to session 1, with the type of questions included depending on the type of participant (see Fig. 2 and the *Baseline* section above).

##### Part 1: Neuropsychological test

Participants complete several neuropsychological tests, including the Matrix reasoning subtest of the WAIS-IV [102] and the Dutch Reasoning Ability Test (*Nederlandse Leesvaardigheidstest voor Volwassenen*; [103]) to assess fluid and crystallized intelligence, respectively. Working memory is assessed using a short version of the Operation Span task.

##### Part 2: Psychoeducation, motivational intervention, goal-setting and planning

Participants receive information regarding the role of adaptive lifestyle behaviors in relation to mental health and well-being and are provided with the intervention rationale: learning a new strategy to promote behavior change in favor of a beneficial lifestyle and good mental health. The strategy group additionally undergoes *psychoeducation* (see Fig. 1) on the role and utility of habit formation in supporting behavior change.

Subsequently, all participants are given a *motivational intervention* (Fig. 1), comprising persuasive information about the benefit of exercise, and walking specifically (e.g., increase cardiovascular fitness, strengthen bones, reduce excess body fat, boost muscle power and endurance, and reduce risks of developing conditions such as heart disease, type 2 diabetes, and osteoporosis). Importantly, it is emphasized that physical activity does not have to be vigorous or done for long periods in order to experience (physical) health benefits, and that we expected them to walk for *a minimum of* 15 min on a daily basis for three weeks, starting on the day after this session. Participants are encouraged to think about why this study is personally relevant to them and what motivated them to sign up. In addition, the strategy group undergoes *psychoeducation* (Fig. 1) on how (and why) IIs can help to improve behavior change.

With the help of the test leader, all participants rehearse a goal intention to support their walking behavior (Fig. 1: *goal-setting*). This goal intention is always ‘I will walk for at least 15 min on a daily basis!’. After that, participants sign a behavioral contract outlining the intention to stick to that goal. This technique is used by health and clinical professionals to commit individuals to making beneficial lifestyle changes [104, 105].

The strategy group also formulates a personalized II (‘If [cue], then I will go outside and walk for at least 15 min.’; Fig. 1: *planning*). Participants are assisted in finding an appropriate event-based cue that occurs on a daily basis (e.g., ‘If I have finished my breakfast, then..’), by letting them describe their behaviors and actions on a typical day and identify those routines that were most consistently performed and therefore provide a good opportunity to act. Once an appropriate cue is identified, the participants are asked to visualize the complete procedure of this event occurring: putting on one’s shoes, going outside, taking a picture, starting walking, and continuing this for at least 15 min. Additionally, they are instructed to consider whether they foresee any obstacles to using the selected cue as initiation point [106–108]. Next, they are informed about the relevance of monitoring and evaluating their plan along the way and are instructed that it is possible to change their if-plan during these evaluation moments.

Finally, all participants are instructed what is expected from them during the subsequent three weeks (i.e., Training phase), with weekly and daily assignments.

##### Part 3: Daily lifestyle monitor (strategy group only)

For individuals from the strategy group, one of the daily assignments is to rate their satisfaction with their lifestyle using the Lifestyle Monitor. This instrument provides a means to determine the right course for a healthy lifestyle by allowing individuals to map their satisfaction (0 – 10) with six different lifestyle categories (i.e., Exercise, Sleep, Nutrition, and Focus & relaxation, Social, Meaning/Purpose) in a two-dimensional hexagon-shaped space. The categories are divided over two classes, with the former three corresponding to ‘health’ and the latter three falling under ‘socio-emotional’. This instrument is introduced to the participants by letting them rate their lifestyle satisfaction for these categories mapped onto this hexagon. Participants are instructed how this Lifestyle Monitor will be used throughout the training phase (in daily questionnaire; see Supplement A), and thereafter (during the second session) to observe potential changes.

##### Part 4: Questionnaires concerning the training phase and goal.

Finally, we assessed participants’ current walking behavior and satisfaction, as well as their motivation, self-efficacy, and expected reward for walking for at least 15 min for the forthcoming weeks.

#### Training

Both the control and strategy group are instructed to work with their goal/plan for 3 weeks. Participants with Android devices are instructed to take a picture with a specific photo app (www.seniorendoenmee.nl/app) every time they enact on their goal (i.e., walked for at least 15 min). This app allows one to take a picture and automatically generates a timestamp that is directly stored on a protected server that can only be accessed by the researchers. The pictures are stored on the individual devices of the participants and include a label at the top (SENIOREN DOEN MEE) and a date stamp at the bottom. Participants with iPhones are instructed to send a message via WhatsApp^2^ (e.g., ‘OK’ or the thumbs-up emoticon) every time they enact on their goal.^3^ Invitations for the weekly and daily questionnaires (strategy group only) are sent automatically via Lotus. For the weekly questionnaires, a reminder is sent the next day at 8 A.M. in case it is not completed yet. If participants encounter any (technical) difficulties or require any form of assistance, they are encouraged to reach out to the test leaders promptly via email or phone.

Performance of the behavior is assessed by asking participants to report on their behavior on a weekly basis (at day 1, 7, 14 and 21, always at 12 P.M.) via an online questionnaire (see *Weekly questionnaire* in *Materials*), including measures of frequency and experienced automaticity, as well as psychological distress and stressor experiences (only GI). The goal intention item of the motivation questions (as included in session 1), as well as plan commitment and execution self-efficacy, perceived goal/plan effectiveness and experienced reward are also presented/assessed. In addition to these questions, participants from the strategy group also undergo a plan- evaluation procedure, where they are guided through three steps that could help them to evaluate the effectiveness of their plan and change whenever necessary (by using the manual). Firstly, participants are encouraged to think about their (walking) behavior (e.g., ‘Have you started to move more?’, ‘Is the plan useful?’, ‘Does it cost you noticeably less effort to go on a walk every day because you have made a concrete plan?’). Secondly, participants are asked to have a critical look at their II and determine whether the plan is still appropriate and helps them to walk on a daily basis, or if it would be better to adjust the plan (Fig. 1: *plan evaluation*). Thirdly, after monitoring and evaluating their plan, participants can decide to keep their current II or modify the if-part of their plan (i.e., selecting a different cue). In case individuals decide to change their II, they have to do so independently and use the knowledge and skills gained during session 1 (Fig. 1: *planning*).

At the end of each day at 5 P.M., participants from the strategy group also receive a text-message (and e-mail if preferred), including a link that provides access to a small set of questions aimed to assess lifestyle satisfaction and hedonic/subjective well-being, in addition to the number of daily hassles and uplifts and their impact on one’s overall level of subjective well-being (see *Daily questionnaire* in *Materials*). Importantly, this daily questionnaire is intended to function as a reflection instrument that helps individuals to identify personal opportunities for reduced stress and improvement of subjective well-being and lifestyle in favor of mental well-being and QoL (Fig. 1: *monitoring*), providing insights for the test phase.

#### Session 2 (1 h)

The second session also takes place online (via Microsoft Teams) and consists of several parts.

##### Part 1

Participants’ satisfaction with their lifestyle will be assessed again. Some questions are asked to establish how often participants were not at home when they usually went for a walk and how often they caught up with it earlier/later that day. Moreover, it is established to what extent the daily questionnaire provided participants from the strategy group with relevant insights on opportunities for reduced stress and what would be the right course for a healthy lifestyle.

##### Part 2

Participants are encouraged to reflect on their previous experience with their goal/plan and think about how this strategy could be applied to other everyday life challenges. Next, participants are instructed to select another everyday life challenge or activity they want to focus on for the subsequent three weeks.

For the control group, this decision-making process is supported by presenting them a list of examples of daily behaviors that may fit their personal needs, with each example corresponding to a specific category of the lifestyle monitor. Additionally, participants are asked which category stands out for them and were they see the most room for improvement. Participants are instructed to select one action from the list of activities/opportunities (or self-invented) corresponding to the category that they want to focus on for the subsequent three weeks. Finally, participants formulate (and rehearse) a goal intention to support their chosen behavior. Participants are instructed to formulate the goal in the same format as used in the first phase of the study (e.g., “I will… on a daily basis.”). Participants are allowed to ask questions in case they are unsure about their decision.

For the strategy group, the selection of another everyday life challenge or activity is supported by providing some tools, and encouraging participants to think about the insights gained through the daily questionnaire, through the following steps:

- Participants are instructed to visualize their lifestyle satisfaction scores on the Lifestyle Monitor in their manual to help them recognize what aspects of their lifestyle could still be improved.
- Participants select one lifestyle category and write down what particular daily actions could improve their satisfaction with this lifestyle category. Participants are presented a list of examples of daily behaviors that may fit their personal needs. Importantly, it is explained that activities may fall within multiple lifestyle categories, rather than one specific category (e.g., meditation may fall within sleep and focus/relaxation).
- Participants are instructed to select one action from the list of activities/opportunities that they want to focus on for the subsequent three weeks. It is emphasized that it can be beneficial to select an action that covers multiple categories (e.g., both exercise and social) or even think about ways how a certain behavior may be adapted in such a way that it fulfills several needs, based on the idea of multifunctionality [109]: gaining and maintaining resources or activities that serve multiple dimensions of well-being simultaneously and in a mutually reinforcing way is of high importance for well-being and/or QoL in later life.
- Next, participants are asked to evaluate whether performing this action would be within their control, whether it would be a realistic action to perform every day, and whether they are (intrinsically) motivated (e.g., doing sports to get in better shape, I find doing sports enjoyable; it gives me a good feeling). In support of this, participants are encouraged to think about the uplifts and resources of good mental well-being (or happiness) based on their daily evaluation during the training phase. Specifically, they are instructed to ask themselves whether the selected action would bring them closer to those uplifting/happy feelings and/or whether they missed out on activities/opportunities that could do so. This overlaps with existing behavioral activation treatment principles were participants have to identify adaptive actions that are personally relevant and rewarding to them, thereby having the potential to restore an adequate schedule of positive reinforcement [16]. In case their personally relevant behavior is not intrinsically motivating, participants are encouraged to think about ways in which performing the behavior could be made rewarding.
- In a similar fashion, participants are instructed to think about daily hassles and sources of stress that they had (frequently) experienced throughout the training phase and are encouraged to reflect on the identified action and see whether that behavior would bring them closer or further away from these stressors, and/or think about activities/opportunities that may help them to reduce these stressors.
- After this critical evaluation of their selection action, participants have to decide whether their selected action/behavior is still considered to be appropriate or that they would rather focus on a different behavior. In case of the latter, participants are encouraged to repeat the critical evaluation steps.
- After successfully completing the evaluation steps, participants formulate (and rehearse) both a goal intention and II to support their chosen behavior. Importantly, goal and plan formulation are left to the participants themselves and individuals do not receive any guidance in selecting an appropriate cue. It is, however, emphasized again that the behavior has to be repeated every single day and that the plan has to be as concrete as possible. Participants are allowed to ask questions if they are unsure about their decision(s).
- Using the Lifestyle Monitor, participants indicate for which categories they expect to experience an improvement throughout the test phase.

Participants are advised to schedule an evaluation moment (at least once a week) to evaluate their plan, according to the steps they have learned during the training phase and described in the manual.

#### Test

Both groups are instructed to work with their new goal/plan for three weeks. The weekly questionnaire comprises similar questions and procedures as described in the training phase. In contrast to the training phase, participants from the strategy group are not guided through the plan-monitoring and evaluation steps, but are simply asked whether or not they have evaluated and changed their plan (and when). Again, at the end of each day, participants from the strategy group receive another questionnaire to assess subjective well-being and perceived energy level, as well as daily hassles/stressors and joys on that day.

#### Session 3 (1 h)

The third session also takes place online (via Microsoft Teams). Similar to the mid-session, after the training phase, some questions are asked to establish how often participants were not at home when they usually performed the behavior. In addition, participants’ mental well-being and QoL, depressive symptomatology, feelings of loneliness, apathy symptoms, satisfaction with lifestyle, inclination to engage in if–then planning, self-efficacy and self-management ability are measured again. Participants from the control group are asked whether they had planned when, where and how they would perform the walking behavior, as well as their chosen behavior for the test phase. Some questions are asked to assess participants’ experiences with the independently generated II/goal and there are some specific questions included about their attitude towards the use of the newly learned strategy in the future.

#### Follow-up (optional)

Participants that are willing to answer some follow-up questions, are sent an additional questionnaire ~ 3 months after the post-session to determine whether they have deployed their newly learned planning strategy for additional personal goals and challenges.

### Materials

An overview of all questions included in the current study can be found in Fig. 2, and a detailed description of all materials is included in Supplement A. Some questions comprise reflection elements, and others are (also) used to evaluate the effectiveness of the MCSI.

## Analyses

All analyses are conducted in R or SPSS. Alpha is set at 0.05 and corrected when specified.

### Aim 1 (a and b): behavioral adaptability and mental health

#### Overview

The first set of analyses focusses on the phase-dependent variables of both the training and test phase and is intended to identify whether the MCSI could successfully support behavioral adaptability by helping individuals to build a new, relatively simple routine of walking every day, as well as a routine of their own choice, (with the latter reflecting the independent and effective use of II as a metacognitive strategy. To this end, two mixed analyses of variance (ANOVA) with Week as within-subject factor (1, 2, 3 or 4) and Group as between-subjects factor (strategy, control) will be conducted for each phase of the study to determine whether significant differences exist among intervention groups and timepoints for behavioral frequency (i.e., total number of walks/personally chosen behavior) and perceived automaticity (self-reported behavioral automaticity index (SRBAI) scores; [40]). Alpha will be Bonferroni corrected for multiple testing (α/2). Subsequently, the potential mediating role of regularity of behavior (i.e., captured in the standard deviation of the timing at which the behavior is performed, with higher scores reflecting lower levels of regularity) on the MCSI effect on perceived automaticity will be assessed through mediation/path analysis, with a SRBAI difference score (day 21 minus day 1) as outcome variable.

In case an overall intervention effect on behavioral frequency and perceived automaticity during the test phase is found, the total behavioral frequency and the SRBAI difference score of the test phase will be included as mediators in another mediation/path analysis (*first step*), with mental well-being and QoL as separate outcome variables. Results of this analyses will be interpreted in a confirmative fashion. Alpha will be Bonferroni corrected for multiple testing (α/2).

In a second step, the phase-independent variables (i.e., self-efficacy, the inclination to engage in if–then planning, lifestyle satisfaction and self-management ability) will be added as potential mediators as well, along with the total behavioral frequency and SRBAI difference score of the training phase. However, in case an overall intervention effect on behavioral frequency and perceived automaticity during the test phase is not found (and step one is skipped), none of the phase-dependent variables will be included in this analysis, including the total behavioral frequency and SRBAI difference score of the training phase even when there appears to be an intervention effect. Again, mental well-being and QoL will be evaluated as separate outcome variables, along with depression, loneliness, and apathy. Results of the second analysis (step) will be interpreted in an exploratory fashion.

#### Assumptions

The assumption of normality, homoscedasticity, and sphericity (for the mixed ANOVAs) will be assessed post-hoc. The assumption of normality will be assessed by plotting the quantiles of the model residuals against the quantiles of a Chi-square distribution for each combination of factor levels (Group vs. Week). In case the quantiles of the residuals do not strongly deviate from the theoretical quantiles and this fall (approximately) along the reference line, normality will be assumed. Homoscedasticity will be evaluated by plotting the residuals against the predicted values. In case points appear randomly distributed across all values of the independent variable, homogenous variance will be assumed. Mauchly’s test will be used to assess the assumption of sphericity [110]. In case of a significant main effect of time or Group x Time interaction effect, (paired) t-test will be conducted to evaluate underlying subgroup differences.

#### Expectations confirmative analyses

**Fig. 3:**
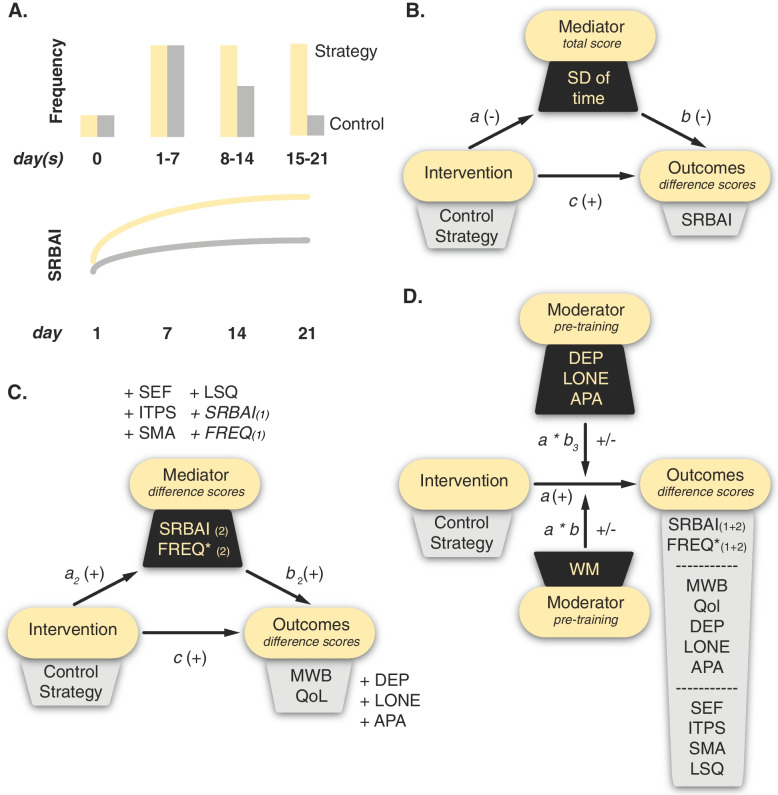
Expectations of our analyses. Panel A illustrates our expectations regarding the frequency and automaticity (as assessed with the self-reported behavioral automaticity index; SRBAI), for the control group (grey) and metacognitive strategy group (yellow) separately. Panel B illustrates the proposed mechanisms through which the metacognitive self-help intervention (MCSI) will exert a positive effect on automaticity, with the regularity of the target behavior as mediator (standard deviation (SD) of the time of the performance of the target behavior). Panel C shows some proposed mechanisms through which the MCSI will exert favorable effects on mental well-being (MWB) and quality of life (QoL), with the total (*) behavioral frequency (FREQ) and SRBAI difference scores of the test phase being included as potential mediators. In a second step, depression (DEP), loneliness (LONE) and apathy (APA) are also included as outcome variables, and self-efficacy (SEF), the inclination to engage in if–then planning (ITPS), self-management ability (SMA), lifestyle satisfaction (LSQ) and FREQ and SRBAI of the training phase as potential mediators. Panel D depicts some proposed moderation effects of the MCSI’s effects on total behavioral frequency and SRBAI difference scores (training and test), as well as MWB, QoL, SEF, ITPS, SMA and LSQ difference scores and DEP, LONE, APA post-study scores. pre-training DEP, LONE and APA, as well as working memory scores (as assessed with the operation-span task) are included as potential moderators. In panel B, C and D, the direction of the anticipated effect is indicated with a + (positive) or – (negative) sign. The control group will be coded as 0 and the strategy group will be coded as 1. The numbers in parentheses indicate whether the variable was derived from the training (1) or test (2) phase, or both

For the phase-dependent variables (see Fig. 3A), we expect overall better scores for individuals in the strategy group. Moreover, perceived automaticity is anticipated to increase over time in both groups, yet stronger among individuals from the strategy group (interaction effect). Behavioral frequency is expected to remain relatively stable among individuals from the strategy group, but to decrease over time among control individuals (interaction effect). Regularity of behavior is expected to be higher among individuals from the strategy group (hence lower standard deviation of time), which is anticipated to positively impact the SRBAI difference score (see Fig. 3B for anticipated, partialmediation effect). Mental well-being and QoL are anticipated to increase, yet steeper for participants from the strategy group (see Fig. 3C). This is reflected in larger absolute difference scores for the strategy group. We anticipate that the intervention’s effects on mental well-being and QoL are partially mediated by perceived automaticity and the total behavioral frequency of test phase.

#### Expectations exploratory analyses

We expect self-efficacy, the inclination to engage in if–then planning, lifestyle satisfaction and self-management ability to have a positive effect on the mental well-being and QoL, as well as the mental health outcomes, as reflected in larger absolute difference scores for the strategy group (Fig. 3C). However, given our sample size constraints, these analyses will be interpreted with more caution, only providing preliminary evidence in case our results meet these expectations.

### Aim 2: underlying mental health (exploratory)

The moderating effect of underlying (pre-training) mental health problems on the effectiveness of the intervention will be assessed by performing several moderation analyses, with moderators being tested individually (see Fig. 3D, *upper moderator panel*). Crucially, for depression, loneliness and apathy as outcome variables, a post-test score rather than difference score will be used to avoid overlap with the mediator. The impact of prior depression, loneliness, and apathy scores on the effectiveness of the MCSI will be assessed in exploratory fashion, with no strong a priori hypotheses on the directionality of the potential moderation effects. Hence, results will be interpreted with more caution, providing only preliminary evidence.

### Aim 3: Working memory capacity (exploratory, two competing hypotheses)

Similarly, the role of working memory capacity on the effectiveness of the MCSI intervention will be explored (see Fig. 3D, *lower moderator panel*).

### Aim 4: general buffer for stress (exploratory)

To assess the effect of the MCSI on the psychological adaptability element of resilience, we investigated the impact of daily stressors/hassles on mental health. To this end, self-reported daily hassles were related to the psychological distress and mood data, derived from the weekly and daily questionnaires.

#### Baseline resilience level (BRL)

A baseline measure of resilience to daily stressors will be quantified to obtain a first indication of participants’ level of resilience to daily stressors. For both the control and strategy group, the relationship between psychological distress (as assessed with the Kessler Psychological Distress Scale, K10; see Supplement A for details) and *stressor exposure* throughout the week prior to the start of the intervention will be assessed first. A prior stressor exposure score will be constructed by combining major life events and daily hassle exposure during the week prior to the intervention. Since the major life events can have an enormous impact, with the consequences lingering for a longer period of time, a *prior MLE* (pMLE) will be quantified *for each MLE* that occurred during the past three months prior to the intervention, using the following equation:

$$\left(baseline\right) pMLE=\frac{{pMLE}_{burden}}{\left(\frac{p{DHs}_{burden}}{pDHs}\right)}$$

with p*MLE*_*burden*_ reflecting the reported burden of that MLE during the week prior to the intervention; pDHs reflecting the total number of *unique* daily hassles experienced during the week prior the intervention; and pDHs_burden_ representing the overall burden of these daily hassles. In other words, the number of unique daily hassles and their overall burden will be used to establish the relative burden per unique daily hassle. In turn, this relative burden per unique daily hassle will be used to infer the weight of the pMLE score, considering its experienced burden. This weighted (baseline) pMLE and pDHs score(s) will be summed to a total prior stressor exposure score. This will be done for each individual separately.

Next, the relationship between psychological distress and stressor exposure during the week prior the intervention will be established. Here, a positive linear relationship between both variables is expected and also serves as a prerequisite for the next steps in determining baseline resilience level. The relationship will be used as reference point, expressing the normative reactivity of psychological distress to stressor exposure in the entire group at baseline. Per individual, the distance of one’s score to this regression will be quantified*.* This residual discloses to what extent the participants deviate from the normal (sample-dependent) relationship. Those having positive residuals are considered to be more resilient, with low stress reactivity, whereas those having negative residuals are considered to be less resilient, with high stress reactivity (i.e., residualization-based calculation of stressor reactivity; see [111]). This baseline measure of resilience to daily stressors will be used in the time course analyses described below.

#### Time courses of stressor reactivity, psychological distress

A similar residualization-based calculation of stressor reactivity procedure will be done for the data collected at the end of the first week of the training phase, only this time for the control and strategy groups separately due to different assessment windows.

For the control group, daily hassle exposure will be assessed on a weekly basis and the total number of hassles experienced during each week will be calculated (wDHs). To take into account the lingering effects of (possible) major life events experienced prior to the intervention period, we will ask participants to report on the experienced burden of their prior MLE at the end of every week. The burden of this pMLE will be divided by the product of the fraction between the number of unique weekly daily hassles (wDHs; denominator) and the overall burden of these weekly hassles (wDHs_burden_; numerator):

$$\left(during\ intervention, weekly\right) pMLE=\frac{{pMLE}_{burden}}{\left(\frac{w{DHs}_{burden}}{wDHs}\right)}$$

The weighted weekly pMLE score will be added to each corresponding weekly wDHs, resulting in the *weekly stressor exposure score*. In case participants report that another, new MLE (nMLE) had occurred, during a weekly questionnaire, the effect of this *nMLE* will also be taken into account for the week of occurrence, as well as the weeks thereafter, using a similar weighted approach.

For the strategy group, daily hassle exposure will be assessed on a daily basis. To obtain an average weekly daily hassle score (wDHs), the total number of reported hassles experienced during each week will be divided by 7. The average weekly burden will be established in an analogous way. Again, the lingering effects of (possible) prior major life events, as well as other, new major life events will be established by using the wDHs, their burden and the reported burden of the pMLE and/or nMLE (see previous equation). The weighted weekly pMLE and nMLE score(s) will be added to each corresponding weekly wDHs, resulting in the *weekly stressor exposure score*.

**Fig. 4:**
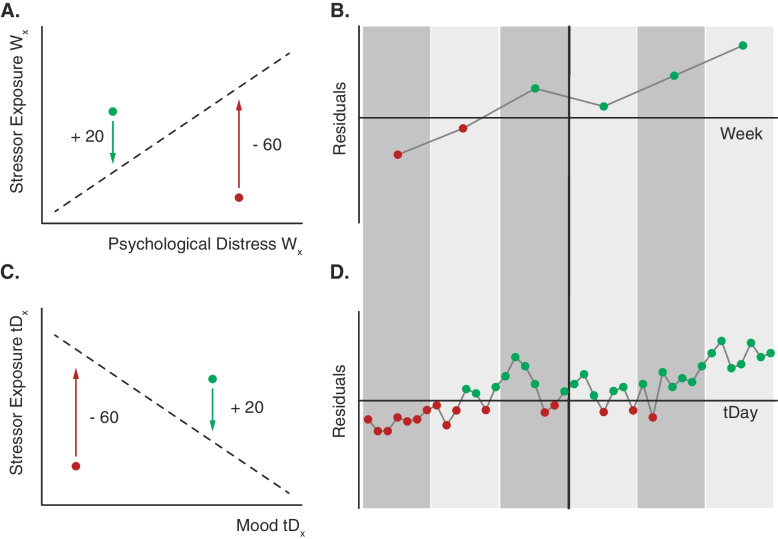
Residualization-based calculation of stressor reactivity. In panel A, the dashed line represents the hypothesized relationship between psychological distress and stressor exposure estimated after the first week (W1) of the intervention. The green and red dots illustrate two example participants, with a negative and positive residual (i.e., distance to the regression line), respectively. A negative residual is considered to reflect low levels of resilience to daily stressors, whereas a positive residual is considered to reflect high levels of resilience to daily stressors. For each subsequent week, individual stressor reactivity scores will be estimated (with x representing the week number) using the reference relationship estimated after the first week. Panel B shows a potential change in stressor reactivity over time. Panel C is analogous to Panel A, only here stressor reactivity is quantified based on the relationship between the average daily stressor exposure and daily mood of the first three days. After that, residuals will be quantified for each subsequent triplet of days (tD), in sliding windows, to shed light on potential changes in stressor reactivity over time (Panel D)

The regression lines of the relationship estimated after the first week (for the control and strategy groups separately), will be used as reference/norm for all the residual calculations in the weeks thereafter to make scores comparable across time (see Fig. 4). Again, a positive linear relationship is anticipated and required.

Subsequently, it will be examined whether psychological distress scores, as well as stressor exposure, show (significant) fluctuations over time. This information is used to better interpret the main analysis, looking at the residuals and their (potential) variation over time. A mixed-effects regression model will be used to analyze the predictive value of time (week), BRL, PASSp and PASSc of the residuals. Specifically, we aim to establish whether (1) the residuals showed meaningful variation over time and are affected by the intervention, (2) whether BRL, PASSp and PASSc affect overall residual scores, and (3) whether these baseline predictors impact the time course of the residuals (interaction effects). The *lmer* function of the R-package *lme4* will be used, with all variables included as continuous fixed-effects predictors. Individual participant ID will be included as a random effect, to allow variation in the intercept among participants. All continuous variables (except for Week) are z-scored prior to model estimations, and a restricted maximum likelihood approach is used. All possible interaction terms will be included.

This analysis will be complemented by a second analysis, including two-third of the participants with the highest overall (sum) stressor exposure throughout the entire intervention period. The first analysis will only be considered to be valid if the results of the second analysis are pointing in the same direction.

#### Time courses of stressor reactivity, daily mood

A comparable, yet adjusted, procedure will be followed for the Mood Questionnaire data that is completed by individuals from the strategy group during the training and test phase on a daily basis. Specifically, to take into account the potential (short) lingering effects of daily hassles, we will construct daily residual scores based on average stressor exposure and mood scores per triplet of consecutive days. The reference regression line will be estimated at day 3, representing the relationship between average stressor exposure and mood of day 1, 2 and 3 together (see Fig. 4). Next, residuals will be quantified for each subsequent triplet of days, in sliding windows (i.e., 2–4, 3–5, 4–6,..). Weighted weekly pMLE and nMLE score(s) will be established in a similar way as previously described for the strategy group, only now an average daily hassle score and their burden will be assessed per triplet of days (tDHs and tDHs_burden_, respectively),

$$\left(during\ intervention, per triplet\right) pMLE/nMLE=\frac{{pMLE/nMLE}_{burden}}{\left(\frac{t{DHs}_{burden}}{tDHs}\right)}$$

These will be added to each corresponding stressor exposure triplet score.

## Discussion

The ability to adapt one's behavior in response to internal and external changes is crucial for resilience. This is especially the case for older adults, who often experience many age-related challenges and transitions. However, as people age, the decline in executive functions can lead to difficulties in voluntary behavior, making it harder for older adults to flexibly adjust their behavior. This can have a negative impact on specific mental health variables, and their quality of life (QoL) and mental well-being in general. Therefore, it is important to develop and provide effective programs that can help older adults to achieve their goals and adapt their behavior as needed. The current study is investigating whether a metacognitive self-help intervention (MCSI) can effectively support behavioral adaptability in favor of resilience among older adults, both with and without subclinical mental health problems. This strategy focuses on gradually automatizing efficient routines to facilitate goal attainment, taking advantage of the fact that habit formation processes tend to remain relatively intact in later life.

This MCSI stands out as an innovative approach to empowering older adults by helping them to set realistic and achievable goals and plans, become more aware of their thoughts, feelings, and behaviors, and identify barriers preventing them from achieving those goals. Unlike traditional programs, our MCSI aims to teach individuals a strategy that they can use independently to set and strive for self-identified goals, enabling them to tackle a multitude of challenges in different domains, not just during but also after the intervention period. This approach contributes to maintaining autonomy and independent functioning and thereby offers a unique and promising way to support behavioral adaptability as vital element of resilience in later life. In addition, by fostering behavioral adaptability, the MCSI may also promote resilience to daily stressors, thereby contributing to successful aging from different angles. Despite these anticipated benefits, there are also some important challenges and potential limitations, which will be discussed below.

### Generalizability to the general older population: potential limitations

Our study aims to obtain results than can be generalized to the entire population. However, as our intervention is delivered partially online, it may not be accessible to all older adults. Some may not have access to technology, or may lack confidence in their knowledge and/ or skills required to use online tools [112, 113]. Moreover, some older adults may be less likely to trust online sources, or may be concerned about privacy, security and data protections [114]. All these factors can give rise to self-selection bias or non-random attrition [115, 116]. In order to minimize potential barriers to participation, we have created a detailed information brochure and a step-by-step guide for participating in our online intervention, and we offer support through email and phone to assist participants with any technical issues they may encounter. Despite these efforts, some participants may face technical problems that cannot be resolved by us, or people may choose not to participate due to a perceived lack of technical proficiency, even before seeking help.

Another issue that could give rise to a certain self-selection bias, is the fact that individuals who sign up for our study are interested in improving their lifestyle, and likely acknowledge that changing one’s behavior is crucial to achieve this. People who do not recognize the importance of a healthy lifestyle and the need to adapt to changing circumstances, may not feel compelled to participate. Notably, the relatively high prevalence of inadequate health literacy (i.e., an individual's ability to read, comprehend, and act on medical instructions) among older adults [117, 118] may impact their willingness to engage in health-promoting behavior. Individual differences in health illiteracy contribute to disparities associated with educational attainment in preventive health behaviors among older adults [119].

Finally, the program’s focus on a pre-determined goal of walking for at least 15 min on a daily basis during the training phase may also deter some individuals from signing up, particularly those who have other specific goals they want to pursue (but not walking per se). We chose this walking-goal for the training phase for several reasons. It is a simple, achievable goal that should be attainable for everyone and can, therefore, boost confidence in participants’ ability to achieve their goals. Having this experience, individuals may also learn how to set their own goals more efficiently. Allowing individuals to set their own goals from the start could result in unrealistic goals. Moreover, the training-test design also allows us to evaluate the importance of the type of behavior selected during the test phase. If individuals successfully develop the routine of daily walking, but do not succeed with their personally formulated goal in the test phase, this could shed light on how well people are able to metacognitively form realistic goals.

Due to these issues/limitations, some older individuals that could actually benefit greatly from our metacognitive self-help strategy, may not sign up and therefore not participate in our study. If we nonetheless are able to demonstrate positive effects of our MSCI, then this provides very strong support for our intervention, and suggests that this is a promising approach to increase behavioral adaptability, and perhaps also resilience to daily stressors, in more vulnerable subgroups.

### Challenges during study: acceptability and compliance

In addition to the challenges that we face with regard to the generalizability of our sample, there are also some challenges that we may encounter during the study. First of all, although older adults may express interest in improving their lifestyle and ability to change their own behavior, they may still resist implementing new strategies that challenge their established habits and routines (e.g., [32]). People may prefer to stay within their comfort zone, especially in later life, which means that they would miss out on the full benefits of the program. This could result in slower progress or limited success in achieving their goals. Additionally, individuals may be skeptical of novel approaches or strategies, particularly if they have had negative experiences with similar programs or attempts in the past. Therefore, an individualized and empathetic approach, providing support and encouragement throughout the program, is essential to build trust, engagement, and increase the likelihood of positive outcomes. Support is provided during online meetings and via e-mail during training and test phases.

Some individuals may resist trying new things or be skeptical about the program due to a lack of self-efficacy or, more specifically, confidence in their ability to change their behavior [120]. Low self-efficacy can reduce goal-directed behaviors and hinder engagement in healthy lifestyles among older adults [121], which may affect their physical and mental functioning. However, our MCSI can empower older adults to set attainable goals and plans, become more aware of their thoughts, feelings, and behaviors, and identify barriers to achieving those goals. This can improve autonomy and self-efficacy, and motivate individuals to actively engage in the program.

Our study also faces a significant challenge in striking a balance between offering an optimal program and effectively evaluating its effectiveness. Most likely, participants are primarily motivated to improve their own well-being and lifestyle. However, some guidelines set forth by the program – to allow for systematic evaluation – may conflict with their own preferences and ideas about how they want to change their behavior (e.g., they do not like the idea of using a strict if–then plan, prefer a passive over an active goal, or would like to focus on a weekly rather than daily behavior). Although we can stress the importance of following the study’s guidelines, some may still choose to pursue their own strategies (e.g., disregarding the if–then plan formulated at the start). Alternatively, compliant individuals may experience some discomfort, leading to reduced involvement. Both can compromise the accuracy of the MCSI's efficacy assessment.

### Alternative interpretations of a potential lack of MCSI effects

Our study aims to determine if our MCSI can support behavioral adaptability in favor of mental well-being, QoL and mental health. We compare a metacognitive strategy group, following the comprehensive MCSI, to a control group, following a limited version of the program which is expected to yield less beneficial results. However, there could be a range of factors that may prevent the strategy group from outperforming the control group.

The key elements that are different between the strategy and control group, is that the strategy group is encouraged to form an II to support their GI (and evaluate their II regularly) and to monitor their daily behavior, feelings, and satisfaction with one’s lifestyle, which can help to identify opportunities for change. While the strategy group may benefit from the depth and comprehensiveness of the program, they may also encounter difficulties in managing the larger volume of information and tasks, which may be overwhelming or difficult to keep up with. On the other hand, the control group may have an easier time remaining engaged and motivated, as the program is easier to manage. In addition, the fact that participants from the control group also engage in elaborate goal setting and diligently monitor their progress towards each goal, may also limit the possibility of identifying additional benefits solely attributable to the MCSI.

Subsequently, there can be several reasons why the benefits of IIs over GIs specifically may be overshadowed. Firstly, an II may not provide any additional benefit over an GI if a superordinate intention is not present or strong enough [34, 35]. This may particularly apply to the training phase, where individuals are instructed to focus on a goal that is introduced by us, and individuals may, therefore, pursue this goal for external reasons (e.g., social pressure). Secondly, although the control group is not encouraged to think about good acting opportunities, they may still do so themselves, thereby spontaneously forming specific plans to support their goals [122]. This could give them an advantage over those directly instructed to formulate an II, as plans generated on one’s own initiative may be easier to remember and enact [83]. In some cases, individuals from the control group may (indirectly) link their behavior to a cue (e.g., ‘Getting out of bed when my husband does’), or chose behaviors/activities that they will only perform at specific times of the day (facilitating temporal regularity; e.g., cycling for 10 min on the exercise bike in the evening). To control for this, during the final questionnaire, individuals from the GI are asked whether they have linked the performance of the behavior to another activity. Thirdly, despite the personalization of individual IIs, some individuals may find a daily plan too rigid and not feasible, for instance due to an irregular lifestyle [123]. While holding specific plans provides numerous benefits, it can also make people inflexible and less likely to act at unplanned times (e.g., when a critical situation is missed, individuals may not easily adapt to a new time or situation; [124] This may provide a disadvantage for the strategy group. Indeed, the strategy group is encouraged to adapt their plan whenever necessary, but some individuals may not directly do so, which can impede behavior change.

Ultimately, the success of the programs may likely depend on individual needs, preferences and abilities of each participant, as outlined in the next section.

### Individual differences

The MCSI may yield positive results for some individuals but not for all, and potential increases in behavioral frequency and automaticity, as well as other variables of interest, on the individual level may be overshadowed by non-changing levels of other participants within the same group. This may be explained by different starting positions (i.e., baseline frequency of a behavior; initial habit strength), with little to no improvement among those that already performed the behavior frequently in the past [74]. The moderating role of baseline mental health and working memory capacity is examined explicitly. However, personality may also play a significant role. Highly hardworking, ambitious, and self- disciplined individuals (i.e. highly conscientious) may, for instance, not experience difficulties with goal striving and thereby not confer extra benefit from our interventions [125, 126]. Additionally, individuals with great regularity in daily social and behavioral rhythms may more easily deploy if–then planning as metacognitive strategy. As data on several personality characteristics are collected as well, such questions could be answered post-hoc, in an exploratory fashion.

### Limited time frame

In accordance with a previous controlled investigation of real-world habit formation, we measured routine automatization for a period of 21 days [127]. While we expect 21 days to be sufficient to demonstrate the short term benefits of the MCSI, especially for easy attainable goals, this short time period means that the present study cannot reveal the effects of the MSCI on lasting, long-term behavior change, which arguably is where the beneficial effects of habit formation should be most apparent. To shed more light on this possibility, we invite participants to complete a follow-up questionnaire approximately three months later to determine if the target behaviors have stuck and to what extent individuals are still using the behavior change strategy that they learned during the present study.

### A more general future outlook

With the current study, we aim to determine whether our MCSI supports behavioral adaptability and mental well-being, QoL and mental health. We have intentionally designed our intervention to be comprehensive and feasible for participants and healthcare providers/coaches, while still potentially providing meaningful benefits. We have drawn inspiration from the proven effectiveness of various behavioral change techniques (e.g., behavioral activation), and have integrated additional self-reflection elements that should encourage individuals to identify patterns, strengths, and lifestyle domains or behaviors that could still be improved. Furthermore, we condensed the intervention into three session and two active phases to strike a balance between effectiveness and practicality. If this intervention indeed offers the anticipated benefits, follow-up research is needed to identify the most crucial components of this intervention and further optimize its effectiveness.

Moreover, to increase the impact of the MCSI, it may be opportune in future steps to stratify the target sample and specifically focus on individuals that have a less healthy lifestyle, lack health literacy, face large challenges in adapting their behavior, and/or are particularly vulnerable (e.g., due to underlying health problems).

Indeed, in the current study we specifically focus on non-clinical and sub-clinical older adults, who do not (yet) require the help of a trained clinician, to examine the potential of our MCSI as an early intervention program. However, our research may also inform the integration of strategic planning as a MCSI as part of (cognitive) behavioral therapy for clinical populations.

### Concluding remark

Despite the challenges and limitations outlined above, our innovative MCSI program offers a promising means to empower individuals and provide older adults with the tools and strategies they need to take control of their goals and plans. By offering a comprehensive and sustainable solution, we aim to help older adults build the skills and confidence they need to thrive in their later years. With a focus on autonomy and independent functioning, our program is uniquely positioned to help older adults stay resilient and adaptable as they face the challenges of aging. In this way, our MCSI has the potential to make a significant difference in the lives of older adults and contribute to healthier and more fulfilling aging.

### Supplementary Information

**Additional file 1. **

## Data Availability

Data sharing is not applicable to this article as no datasets were generated or analysed during the current study.

## Abbreviations

ANOVA: Analysis of variance
BRL: Baseline resilience level
DH: Daily hassle
GI: Goal intention
II(s): Implementation intention(s)
K10: 10-Item Kessler Psychological Distress Scale
MCSI: Metacognitive self-help intervention
nMLE: New major life events
PASSp: Positive appraisal style scale, problem focused
PASSc: Positive appraisal style scale, content focused
pMLE: Previous major life events
QoL: Quality of life
SRBAI: Self-reported behavioral automaticity index
tDHs: Average number daily hassles per triplet of days
tDHsburden: Average burden of daily hassles per triplet of days
wDHs: Number of unique weekly daily hassles
wDHsburden: Burden of weekly daily hassles

## Footnotes

1 To illustrate, a three parallel mediation model with similar settings resulted in an estimated power of 0.64 to 0.67.

2 Participants’ phone numbers are stored under a unique participant number, and are not linked to their name/e-mail address.

3 To ensure anonymity, iPhone users are instructed that they can only use WhatsApp to communicate with us when they enact on their goal. For other queries, they can reach out to us via e-mail.
