# Supplementary material for: Improving goal striving and resilience in older adults through a personalized metacognitive self-help intervention: a protocol paper
Source: BMC Psychol. 2023 Aug 4;11:223. doi: 10.1186/s40359-023-01259-3 (PMC10403928; doi:10.1186/s40359-023-01259-3)
Supplement: Supplementary file 1 — Additional file 1. [file 40359_2023_1259_MOESM1_ESM.pdf]

[SUPPLEMENT A: MATERIALS] Improving Goal Striving and Resilience Through a Personalized Metacognitive Self-Help Intervention in Older Adults.

Lotte P. Brinkhof[a,b,c]*, K. Richard Ridderinkhof [a,b,c], Jaap M.J. Murre[a,b,c], Harm J. Krugers[b,c,d], Sanne de Wit[a,b,c]

[a]*Dept. of Psychology, Faculty of Behavioural and Social Sciences, University of Amsterdam, Amsterdam, Netherlands;*
[b]*Centre for Urban Mental Health, University of Amsterdam, Amsterdam, Netherlands;*
[c]*Amsterdam Brain & Cognition (ABC), University of Amsterdam, Amsterdam, Netherlands*
[d]*Faculty of Science, Swammerdam Institute for Life Sciences, University of Amsterdam, Amsterdam, Netherlands*

*Lotte. P. Brinkhof, E-mail: l.p.brinkhof@uva.nl, Address: Nieuwe Achtergracht 129, 1001 NK Amsterdam, Netherlands.

Questions and tests prior to session 1

Inclination to engage in if-then planning

The If-Then Planning Scale (ITPS) of Bieleke and Keller [1] was used to determine one's tendency to engage in if-then (or implementation intention) planning. The scale comprises eight items in total. The first four items relate to the identification of critical situations (if-part), the other four items covered aspects of the then-part (i.e., specifying goal-directed behavior). One half of the items focussed on seizing opportunities (e.g., 'I think about when and where decisive moments for the achievement of my goals could occur'), and the other half related to dealing with obstacles (e.g., 'I envisage what obstacles could arise'). Answers were scored on a 7-point Likert Scale (1 = does not apply at all, 7 = does fully apply) and summed to a total score, with high values reflecting a higher tendency to engage in if-then planning.

Conscientiousness

The NEO-FFI conscientiousness sub-scale (12 items) was used to assess individual differences in the personality dimension conscientiousness [2]. Each item (e.g., "I keep things neat and clean') was scored on a 5-point Likert scale (1 = strongly disagree, 5 = strongly agree). The four negatively formulated items were reverse scored, and a total sum score ranging from 12 - 60 was computed. Higher scores indicated more conscientiousness.

Lifestyle regularity

An adaptation of the short Social Rhythm Metrix (SRM-5) was used to quantify daily lifestyle regularity [3,4]. The original SRM-5 is a diary-like questionnaire, in which participants are supposed to report on the timing of five specific events (e.g., getting up in the morning) on a daily basis for one week. Based on these data, a habitual time for each event is calculated and the number of occurrences within a 1.5-hour window around this habitual time is counted to determine the regularity of that event. The combination of these evens together is supposed to reflect one's lifestyle regularity. To reduce the burden on participants, we did not ask individuals to fill out the questionnaire on a daily basis, but instead asked them to indicate the timing on which these events usually took place in the previous month (i.e., habitual time). Subsequently, they were instructed to report on approximately how many days in the past two weeks the events occurred within a 1.5 hour window (45 min before to 45 min after the habitual time), and outside this window. 'Having lunch' was added to the list of events, resulting in regularity scores for 6 events in total.

Prospective Memory

The short Metacognitive Prospective Memory Inventory (MPMI-s; [5]) was used to assess prospective memory ability, the use of mnemonic (internal) strategies, and the use of external memory aids to remember intentions. The MPMI-s comprises 24 items, divided over three scales. The Prospective Memory Ability (PMA) scale taps onto abilities, with some referring to prospective remembering (e.g., 'I remember my appointments which are coming up in a few days without writing them down.') and others to prospective forgetting (e.g., 'I forget to call a friend again after I could not reach him or her on the first try.'). The *PMSi* scale measures the frequency with which people use internal strategies to remember their intentions (e.g., 'In my mind, I make a list of things that I still have to complete.'); the *PMSe* scale assesses how frequently people use external strategies to remember intentions (e.g., 'I keep a calendar with all of my appointments.'). All items were scores on a 5-point Likert-scale (1 = rarely, 5 = often), and items relating to prospective forgetting were reverse scores before subscale summation. Higher subscale scores (8 – 48) were indicative of better PM abilities or more frequent use of (internal or external) strategies.

Mental well-being

The Dutch language version of the 14-item Warwick-Edinburgh Mental Wellbeing scale (WEMWBS; [6], as originally created by Tennant and colleagues [7] was used to assess mental well-being. The scale contains 14 positively worded items that address aspects of positive mental health (e.g., 'I have been feeling good about myself') over the past two weeks. Each item was scored on a 5-point Likert scale (1 = never , 5 = always) and summed to a total of 14 to 70 points, with higher scores indicating better mental well-being.

Quality of Life
The Dutch language version of the World Health Organization Quality of Life Instrument-Older Adults Module (WHOQOL-OLD; [8,9]) was used to assess quality of life (QoL) over the past two weeks. The measure consists of 24 items, comprising six subscales of four items each: *sensory abilities* (SAB; e.g., 'To what extent do impairments to your senses (e.g., hearing, vision, taste, smell, touch) affect your daily life?'), *autonomy* (AUT; e.g., 'How much freedom do you have to make your own decisions?'), *past, present and future activities* (PPF; e.g., 'How satisfied are you with what you have achieved in life?'), *social participation* (SOP; e.g., 'To what extent do you feel that you have enough to do each day?'), *death and dying* (DAD; e.g., 'How scared are you of dying?'), and *intimacy* (INT; e.g., 'To what extent do you feel a sense of companionship in your life?'). Items were scored on 5-point Likert scales, with different wording, and summed across each subscale. A total QoL score was also calculated, with higher scores indicated better QoL. Some items were reverse scored prior to summation.

Depression
The 15-item Geriatric Depression Scale (GDS; [10–12]) was used to assess depressive symptomatology over the past two weeks. The GDS-15 consists of 15 questions that can be answered with 'yes' or 'no' (e.g., 'Do you feel that your life is empty?'). One point was assigned to each answer that was indicative for depressive symptomatology, and a total sum score was calculated (range: 0 – 15).

Loneliness
The 11-item Loneliness Scale (LS), as developed by de Jong-Gierveld and van Tilburg [13], was used to assess current (based on the past two weeks) overall loneliness, as well as emotional and social loneliness. The scale consists of 11 items, with six items being formulated negatively (emotional subscale) and 5 items positively (social subscale). Possible answers are: "yes!", "yes", "more or less", "no", "no!". Scores were determined by counting the number of neutral and positive answers on the positively formulated items (emotional loneliness score) and counting the number of neutral and negative answers on the negatively worded items (social loneliness score). A total loneliness score was computed by taking the sum of the emotional loneliness score and the social loneliness

Apathy Evaluation Scale (self-Rated)
Apathy was assessed using the 18-item Apathy Evaluation Scale (Self-Rated; AES-S; [14], which has been shown to be valid and particularly reliable among older individuals that are cognitively normal [15]. Each item was scored on a 4-point scale, ranging from (3) not at all true to (0) very true for positive items (e.g., 'I am interested in having new experiences.') and (0) not at all true to (3) very true for negative items (e.g., 'I put little effort into anything.'). Items scores were summed to a total ranging from 0 to 54, with higher scores being indicative of

Psychological distress
The 10-item Kessler Psychological Distress Scale (K10) was used to measure psychological distress [16,17]. The K10 consists of 10 negatively formulated items, each assessing the severity of a mental problem over the past *week* using a 4-point scale: (4) 'all of the time', (3) 'most of the time', (2) 'some of the time', (1) 'a little of the time', (0) 'none of the time'. Scores were summed to a total ranging from 0 to 40 and higher scores were indicative of more psychological distress.

Prior major life events
Participants were also asked whether they had experienced any major life event (MLE) during the *past three months* (e.g., losing a beloved person, divorce, accident), prior to the intervention. For each selected MLE, the extent to which it was burdensome to them during the *past week* was assessed using the following scale: (1) 'not burdensome at all', (2) 'barely burdensome', (3) 'somewhat burdensome', (4) 'burdensome', (5) 'very burdensome'. A MLE score was constructed based on personal evaluations of the severity of daily hassles, which is explained in more detail in the Analyses section of the main article.

Daily Hassle Exposure

Exposure to unpleasant daily experiences/situations (hassles) during the week before the intervention study was examined using a compiled list of hassles based on other hassle scales (e.g., [18–20]). This list comprised hassles that were particularly relevant for older adults. To reduce the burden of participants, several items targeting similar problems (e.g., feeling useful, appreciated; being criticized, ignored, discriminated, etc.) were combined, resulting in a total of 25 hassles. Participants reported whether each hassle had taken place during the past week and how burdensome it was altogether using the same scale as used for the major life events. The total number of daily hassles (count) constituted the daily hassle score (DHs).

Lifestyle satisfaction

Satisfaction with lifestyle was determined for all categories (i.e., Exercise, Sleep, Nutrition, Focus & relaxation, Social, Meaning/purpose) of the Lifestyle Monitor separately by asking participants to rate their satisfaction for each of them (hereafter referred to as the Lifestyle Satisfaction Questionnaire; LSQ). Response categories ranged from 0 (unsatisfied) to 10 (satisfied), and both category-specific and overall LSQ scores were calculated. Additionally, the importance of having a healthy lifestyle, as well as the level of motivation to accomplish this, was assessed separately using a scale ranging from 0 (not important/motivated at all) to 5 (extremely important/motivated).

Self-efficacy

The General Self-Efficacy Scale was used to assess how one generally copes with stressors or challenging situations in life [21,22]. It concerns ten statements that ask how people generally think and act, focussing explicitly on a person's self-confidence that his or her actions are responsible for successful outcomes or that they have control over challenging demands of the environment. Each statement is scored on a 4-point Likert Scale (1 = not true at all, 2 = hardly true, 3 = moderately true, 4 = exactly true) and summed to a total score of 10 to 40, with higher scores reflecting higher self-efficacy.

Self-management ability

The Self-Management Ability Scale (SMAS-18; [23]) was used to measure self-management ability (SMA). The 18-item version of the original 30-item SMAS [24] consists of six three-item subscales. Items corresponding to the *taking initiative*, *investing*, and *positive frame of mind* subscales were scored on a 6-point Likert scale (1 = never, 6 = very often). Another 6-point scale, with 1 = none, 2 = one, 3 = two, 4 = three or four, 5 = five or six and 6 = more than six, was used to score the items corresponding to *variety* subscale. Items corresponding to the *multifunctionality* subscale were scored on a 5-point Likert scale ranging from strongly disagree (1) to strongly agree (5). Finally, items corresponding to the self-efficacy subscale were scored on a 5-point Likert scale ranging from I am certain that I cannot (0) to I am completely certain that I can (5). Scores were calculated by recoding the scores to 0-5 or 0-4 (for the 6 and 5-point scales, respectively) and multiplying the items with 6 options by 4 and the items with 5 options by 5. After that, subscale scores were determined by taking the average of all items corresponding to each scale and multiplying that score by 5. Hence, subscale scores range from 0 to 100, with higher scores reflecting higher SMA in that dimension. SMAS total scores were calculated by taking the average of all mean subscale scores. Here, higher scores indicated higher overall SMA.

Positive Appraisal Style

Two positive appraisal style scales were used to assess the typical thinking processes when encountering a stressor (process focussed, PASSp) and the typical content of an individual's thoughts when challenged (content focussed, PASSc). The 14-item PASSp was developed based on a factor analysis [25] on the subscale level of the brief COPE [26], CERQ-short [27], and two additional items on distanced stressor appraisal. The final measure includes subscales from both the brief COPE (two items), CERQ short (eight items) and the two self-generated items (on distanced stressor appraisal), and reflects positive appraisal content and processes based on seven subscales (i.e., distanced stressor appraisal, positive reappraisal, acceptance, putting into perspective, refocus on planning, positive refocusing and humor). The 12-item PASSc was constructed by evaluating 29 self-generated items on positive appraisal style, with 12 items loading to one clear factor (content; [28]). Participants filled out both the PASSp and PASSc. The two PASSp humour items (including, e.g., "I've made a joke about it"), derived from

the shortened COPE were scored on a 4-point Likert Scale (1=not at all, 2=a little bit, 3=quite a lot, 4=a lot), whereas the other 12-items (e.g., "I think that I have to accept that this has happened") were scored on a 5-point Likert scale [1=(almost) never, 2=sometimes, 3=regularly, 4=often, 5=(almost) always]. The PASSc items were scored on a 4-point Likert scale [1=never, 2=sometimes, 3=often, 5=almost always].The PASSp score was determined by taking the average of the z-normalized scores of the items derived from the shortened COPE, the CERQ, and the self-generated items. The items from the PASSc were summed to a total score of 12 – 48.

Working Memory
Working memory was assessed with a shorter version of the Operation Span task [29]. Participants had to remember a sequence of words in the correct order while simultaneously solving simple mathematical equations. In this version (based on [30]), the number of equations and words per set varied between three and five, and each set type was presented three times (nine in total). The average percentage correctly remembered words (at the correct location) were computed per set type and all set-scores were averaged into a total score [31].

Online session 1 (start) – Questions following planning
Current walking behavior
Participants answered the following question, 'How much do you walk during a typical week?', using eight response categories ranging from (0) never to (7) every day/seven days per week.

Satisfaction with current level of walking behavior
Satisfaction with walking behavior was examined by asking participants to rate their level of satisfaction with the number of (short) walks that they make on a weekly basis on a scale from 0 (unsatisfied) to 10 (satisfied).

Goal
Motivation for walking for at least 15 minutes for the forthcoming 3 weeks was measured using Theory of Planned Behavior items (TPB; [32]), as measured with 7-point scales ranging from 1 (completely disagree) to 7 (completely agree), with higher scores indicating cognitions more favourable to walking: intention, attitude, subjective norms, perceived behavioral control. In addition, two items concerning self-efficacy and expected reward were added as well. *Intention/goal commitment:* 'I plan/want/am motivated to walk for at least 15 minutes on a daily basis' (three items). *Attitude:* 'Walking at least 15 minutes on a daily basis is good for me.' *Perceived social norms (subjective norms):* 'The people in my life whose opinion I value would approve of me walking 15 minutes on a daily basis.' *Control perceptions (perceived behavioral control):* 'I believe that I have control over whether or not I will walk for 15 minutes every day'.). *Self-efficacy:* 'I am confident that I will be able to walk for at least 15 minutes on a daily basis.' *Expected reward:* 'I expect to enjoy walking for at least 15 minutes on a daily basis'.

Secondary goal (Strategy group only)
Participants from the strategy group indicated whether they had formed their II in such a way that it can help to break undesired patterns of behavior (e.g., going for a walk at the time one usually watched TV).

Training
Daily behavior
Frequency
The number of times (or *frequency*) each participants went for a walk each week, and during the complete training phase, was calculated by evaluating the registered number and dates of the pictures/WhatsApp messages that were taken/send. If participants took more than one picture, or send more than one message, on the same day, this was counted as one walk. Participants could indicate if they had forgotten to take a picture/send a message during a walk at the end of each week (see Weekly Questionnaire), which was used to adjust the frequency. Yet, participants were strongly encouraged to take pictures/send messages to ensure high accuracy.

Regularity
As a measure of intake (ir)regularity, we calculated the standard deviation of the registered times for the complete training phase. Regularity scores were not adjusted based on manual input from participants in case they had forgotten to take a picture/send a message and were thus considered as missing values. In case participants indicated to have changed the if-part of their plan (e.g., different cue) during one of the weekly questionnaires, regularity was assessed for registered times before and after this adaption. Next, an overall regularity score was calculated by taking the average value.

Daily Questionnaire (Strategy group only) – 2 to 5 minutes
Lifestyle satisfaction
Lifestyle satisfaction was assessed by using two questions targeting two classes of the Lifestyle Monitor separately: "All things considered, how satisfied were you today with your lifestyle within the categories corresponding to the health/socio-emotional class of the Lifestyle Monitor?". Answers were scored on a scale ranging from 1 (unsatisfied) to 10 (satisfied). It was emphasized that some categories within these classes may seem more important than others, which may even differ per day, and that the answer should therefore reflect a general score of satisfaction per class. Participants were allowed to determine for themselves how much each category would weigh in their class assessment.

Subjective well-being
Subjective (or hedonic) well-being (SWB) was determined by evaluating its affective and cognitive component [33]. The *affective* component of SWB refers to the frequency and intensity of positive and negative emotions/mood and was assessed by asking participants to indicate how they felt on the 6-item mood scale of Wilhelm and Schoebi [34]. This scale is based on the Multidimensional Mood Questionnaire (MMQ; [35] and was explicitly developed to use for momentary assessment and capture temporal variations. The items were preceded with "Today, I felt", and were followed by six mood dichotomies corresponding to basic affective states of valence (V, i.e., content-discontent, unwell/well), calmness (C, agitated-calm, relaxed-tense) or energetic arousal (E, i.e., tired/fatigued-awake, full of energy-without energy). One additional item was added to assess happiness specifically: unhappy/happy. Each item was scored on a scale ranging from 0 to 6, with the three negatively formulated items being reverse scored such that higher scores indicated higher positive V, E, C, happiness or overall mood. The *cognitive* component of SWB was subsequently covered by using a simple question targeting life satisfaction: 'All things considered, how satisfied were you with your life today on a scale of 1 (unsatisfied) to 10 (satisfied).

New major life events
Participants were asked whether they had experienced any (new) major life event since the last completed daily questionnaire (e.g., losing a beloved person, divorce, accident). If yes, participants were asked whether this event had overshadowed every other daily hassles, such that they were irrelevant and did not matter anymore. If yes, participants would not be asked to fill out the daily hassles and uplifts scale. How burdensome this new major life events is, was established during the weekly questionnaire.

In case participants previously indicated to have experienced a major life event during the study period and reported that all daily hassles were overshadowed by this event in the preceding measurement, they were asked whether this was still the case. If yes, participants would still not be asked to fill out the daily hassles and uplifts scale, and were also not asked whether any other life event occurred since the last completed daily questionnaire. However, this latter question was asked to the participants that had experienced a new major life event during the study period already, but indicated this did not (anymore) overshadow all of their daily hassles in an earlier measurement. The participants also filled out the daily hassles and uplifts scale, unless a new, additional major life events had (again) overshadowed their daily hassles.

Daily hassles and uplifts exposure (conditional, see new major life events)
Exposure to daily hassles during the past 24 hours was assessed using the same 25-item list, as well as scoring categories, as previously described. In addition, exposure to pleasant experiences/situations (uplifts) was also examined. Again, a list of uplifts that were particularly relevant for older adults was constructed based on previous scales (e.g., [18–20]). Items were combined when appropriate, resulting in a total list of 17 uplifts. Participants reported whether each uplift had taken place during that day and indicated how uplifting these were altogether using the following categories: (1) 'not uplifting at all', (2) 'barely uplifting', (3) 'somewhat uplifting', (4) 'uplifting', (5) 'very uplifting'. A total count, as well as weighted score (count * how uplifting) was calculated.

Tipping point
Individuals who scored a 2 or lower on the happiness item of the mood scale were also asked to complete the two-item Tipping Point Index [36] to identify when participants' coping mechanisms may have been on the point of breaking down, potentially being in close proximity of a point of tipping into a state of mental disease (i.e., depression and/or anxiety). Participants had to indicate whether or not (0 or 1) they had noticed any of the following reactions over the last week: 'Felt complete hopelessness about the future' and 'Been often feeling panicky or on the point of losing control of your emotions'. Prediction of severe anxiety or depression was based on a yes to either or both of the items.

Weekly Questionnaire
No performance
Participants could indicate if they could not walk because of external/personal reasons (e.g., physical problems). In case participants expected their personal problems would also withhold them from enacting on their plan in the upcoming week, they were asked to contact us.

Picture/WhatsApp message forgotten
In case participants had forgotten to take a picture/send a WhatsApp message during a walk, they could provide the date(s) manually, such that the frequency measure could be adapted.

Automaticity
The Self-Report Behavioral Automaticity scale (SRBAI; [37]; automaticity subscale of the SRHI), was used to assess the experienced automaticity of both walking and taking a picture/sending a WhatsApp message. The behavior of interest was followed by four statements to which participants reported their level of agreement (e.g., 'Walking for at least 15 minutes on a daily basis.. is something I do without thinking'), using a visual analogous scale from 0 – 100 (strongly disagree – strongly agree). On day 1, we specified that these statements referred to walking and taking a picture/sending a message 'so far'; for the other days, it referred to walking 'in the last week'. A mean SRBAI score was calculated for each behavior, with higher scores indicating stronger automaticity. Scores were considered to be missing when the SRBAI was completed too early, more than 2 days too late, or not at all. Participants missing the first and final measurement (at day 1 and 21) were excluded from all further analyses. Missing values in subsequent weeks (at day 7 and 14) were imputated for this analysis using linear interpolation. An SRBAI difference score (day 21 *minus* day 1) was also computed.

Motivation, attitude, reward
Motivation (item from intention questions) and attitude were assessed similar to online session 1. Experienced reward was assessed with a similar item as previously reported, only in the present tense ("I enjoy walking for at least 15 minutes on a daily basis') and not on day 1.

Plan (Strategy group only)
To check whether participants had been working with the plan that they formulated during the first online session, participants were asked the following question: 'Which plan did you formulate to encourage walking on a daily basis?'

Perceived plan effectiveness (Strategy group only)
Perceived plan effectiveness was examined with two items ('This plan helped me to walk on a daily basis', 'This plan helped me to walk at a specific moment of the day'), on 7-point scales from 1 (completely disagree) to 7 (completely agree).

Prior major life events (not at day 1)
Participants that previously indicated to have experienced a MLE throughout the past three months were again asked to what extent this MLE was burdensome to them the past week. In case participants reported any new MLE in the previous weekly assessments (see below), this event was also included here.

New major life events (not at day 1)
Participants were also asked whether there had been any new MLE throughout the past week and how burdensome this was to them.

Psychological distress (control and strategy group) and daily hassle exposure (control group only) was measured similar to previous assessments with a timeframe of one week (both not at day 1).

Online session 2 (mid) – Questions and tests prior to planning
Satisfaction with their lifestyle was measured similar to previous assessments.

Walking if not home
The number of days that participants were not at home at the time they usually went for a walk, as well as the number of days that people still went for a walk at a different moment on these days was examined to better interpret their behavior, as well as missing days.

Secondary goal evaluation (Strategy group only)
If participants indicated that they had formulated their plan in such a way that it would interrupt undesired patterns of behavior, they were asked whether their plan indeed helped to accomplish this.

Future of daily walking
Participant's motivation to continue walking on a daily basis was assessed by asking their level of agreement with the following statement: 'I am motivated to continue walking on a daily basis after the first phase of the study', using a 7-point scale ranging from 1 (completely disagree) to 7 (completely agree).

Habit experience
The extent to which the act of walking on a daily basis was considered to be a habit, was assessed by asking participants to give a score on a VAS scale, ranging from 0 (not at all) to 100 (very strongly).

Experiences with daily questionnaire (Strategy group only)
The extent to which the daily questionnaire provided participants from the strategy group with relevant insights their (1) daily routine/lifestyle and sources of (2) happiness and (3) unhappiness/stress was examined by asking participants to rate their level of agreement with the three statements (e.g., 'The daily questionnaire gave me insights about the things that make me happy'), on a 7-point scale ranging from 1 (completely disagree) to 7 (completely agree).

New major life events
Participants were also asked whether there had been any new MLE throughout the past few days, since the last weekly questionnaire of the first phase of the study.

Online session 2 (mid) – Questions following planning
Participants completed the same questions as were asked prior to the training phase, with all questions directed at their personal, newly chosen behavior instead of walking. In addition, participants had to

enter their goal and plan (Strategy group only) to direct the questionnaires to their newly chosen behavior and evaluate the quality of their plan.

Test

Daily behavior

Daily behavior was assessed similar to the training phase.

Daily Questionnaire

The daily questionnaire of the test phase was similar to the daily questionnaire of the training phase.

Weekly Questionnaire

The weekly questionnaire of the test phase also comprised similar questions to the weekly questionnaire of the training phase. Here, all questions were directed to their personal, newly chosen behavior instead of walking. The burden of all previously reported major life events prior as well as during the study was assessed.

Online session 3 (post)

Mental well-being and quality of life, depressive symptomatology, feelings of loneliness, apathy, satisfaction with lifestyle, inclination to engage in if-then planning, self-efficacy, self-management, as well as the performance of the behavior when not home, secondary goal accomplishment, future of the chosen behavior and habit experience were measured similar to previous assessments.

Spontaneous planning (control group only)

The control group also completed some questions to assess whether they spontaneously formulated specific plans to support their behavior. Each question was asked twice: for walking (training) and their personally chosen behavior (test). Participants were asked whether (1) they performed the behavior at a specific moment during the day, (2) they related the behavior to a specific daily activity or moment, and (3) they formulated a plan in advance to perform the behavior at a specific moment/after a specific activity during the day. Answers were either ‘yes’ or ‘no’.

Current walking behavior

Participants answered the following question, ‘On average, how many days a week did you walk throughout the past week?’, using eight response categories ranging from (0) never to (7) every day/seven days per week.

Experiences and future use

Firstly, participants were asked to rate their level of agreement with four statements related to usefulness of this intervention study (e.g., “The strategy I became acquainted with during this study.. has helped me to perform a daily behavior / has given me more control over my daily routines and lifestyle / has improved my well-being/mental satisfaction / I would recommend to my loved ones”) on a scale from (1) ‘completely disagree’ to (7) ‘completely agree’. Secondly, using a similar scale, participants rated their level of agreement with four statements related to their intentions to continue performing the behaviors of interest (e.g., “The first goal I worked on during this study (going for a daily walk), I want to continue to pursue.”) and/or to use the strategy in the future (e.g., “I plan to use the new strategy I learned more often in the future, for other activities/behaviors.”). Thirdly, participants indicated how much they had learned from the intervention program (all things considered) on a scale of (1) ‘learned little’ to (10) ‘learned a lot’.

References

[1] Bieleke M, Keller L. Individual differences in if-then planning: Insights from the development and application of the If-Then Planning Scale (ITPS). Pers Individ Dif 2021;170:110500. https://doi.org/10.1016/j.paid.2020.110500.

[2] Costa PT, McCrae RR. NEO five-factor inventory (NEO-FFI). Odessa, FL Psychol Assess Resour 1989;02.

[3] Monk TH, Flaherty JF, Frank E, Hoskinson K, Kupfer DJ. The social rhythm metric: An instrument to quantify the daily rhythms of life. J Nerv Ment Dis 1990;178. https://doi.org/10.1097/00005053-199002000-00007.

[4] Monk TH, Reynolds Iii CF, Machen MA, Kupfer DJ. Daily Social Rhythms in the Elderly and Their Relation to Objectively Recorded Sleep. vol. 15. 1992.

[5] Rummel J, Danner D, Kuhlmann BG. The short version of the Metacognitive Prospective Memory Inventory (MPMI-s): factor structure, reliability, validity, and reference data. Meas Instruments Soc Sci 2019 11 2019;1:1–8. https://doi.org/10.1186/S42409-019-0008-6.

[6] Ikink JGM, Lamers SM, Boliuer JM. De Warwick-Edinburgh Mental Well-being Scale (WEMWBS) als meetinstrument voor mentaal welbevinden in Nederland. 2012.

[7] Tennant R, Hiller L, Fishwick R, Platt S, Joseph S, Weich S, et al. The Warwick-Edinburgh mental well-being scale (WEMWBS): Development and UK validation. Health Qual Life Outcomes 2007;5:63. https://doi.org/10.1186/1477-7525-5-63.

[8] Gobbens RJJ, van Assen MALM. Psychometric properties of the Dutch WHOQOL-OLD. Health Qual Life Outcomes 2016;14:1–9. https://doi.org/10.1186/s12955-016-0508-5.

[9] Power M, Quinn K, Schmidt S. Development of the WHOQOL-Old module. Springer 2005;14:2197–214. https://doi.org/10.1007/s11136-005-7380-9.

[10] Burke WJ, Roccaforte WH, Wengel SP. The short form of the Geriatric Depression Scale: a comparison with the 30-item form. J Geriatr Psychiatry Neurol 1991;4:173–8. https://doi.org/10.1177/089198879100400310.

[11] Sheikh JI, Yesavage JA. Geriatric Depression Scale (GDS): recent evidence and development of a shorter version. Clin Gerontol J Aging Ment Heal 1986.

[12] Yesavage JA, Brink TL, Rose TL, Lum O, Huang V, Adey M, et al. Development and validation of a geriatric depression screening scale: A preliminary report. J Psychiatr Res 1982;17:37–49. https://doi.org/10.1016/0022-3956(82)90033-4.

[13] De Jong-Gierveld J, Van Tilburg T. Manual of the Loneliness Scale. Fsw.vuNl 1999:1–26.

[14] Marin RS, Biedrzycki RC, Firinciogullari S. Reliability and validity of the Apathy Evaluation Scale. Psychiatry Res 1991;38:143–62.

[15] Guercio BJ, Donovan NJ, Munro CE, Aghjayan SL, Wigman SE, Locascio JJ, et al. The Apathy Evaluation Scale: A Comparison of Subject, Informant, and Clinician Report in Cognitively Normal Elderly and Mild Cognitive Impairment. J Alzheimer’s Dis 2015;47:421–32. https://doi.org/10.3233/JAD-150146.

[16] Kessler RC, Andrews G, Colpe LJ, Hiripi E, Mroczek DK, Normand SLT, et al. Short screening scales to monitor population prevalences and trends in non-specific psychological distress. Psychol Med 2002;32:959–76. https://doi.org/10.1017/S0033291702006074.

[17] Kessler RC, Barker PR, Colpe LJ, Epstein JF, Gfroerer JC, Hiripi E, et al. Screening for serious mental illness in the general population. Arch Gen Psychiatry 2003;60:184–9. https://doi.org/10.1001/archpsyc.60.2.184.

[18] Brantley PJ, Waggoner CD, Jones GN, Rappaport NB. A daily stress inventory: Development, reliability, and validity. J Behav Med 1987;10:61–73. https://doi.org/10.1007/BF00845128.

[19] Chmitorz A, Kurth K, Mey LK, Wenzel M, Lieb K, Tüscher O, et al. Assessment of microstressors in adults: Questionnaire development and ecological validation of the mainz inventory of microstressors. JMIR Ment Heal 2020;7:e14566. https://doi.org/10.2196/14566.

[20] Kanner AD, Coyne JC, Schaefer C, Lazarus RS. Comparison of Two Modes of Stress Measurement: Daily Hassles and Uplifts Versus Major Life Events. J Behav Med 1981;4.

[21] Schwarzer R, Jerusalem M. General Self--efficacy Scale (GES). 1995.

[22] Teeuw B, Schwarzer R, Jerusalem M. Dutch adaptation of the general self-efficacy scale. Berlin, Ger 1994.

[23] Cramm JM, Strating MMH, de Vreede PL, Steverink N, Nieboer AP. Validation of the self-management ability scale (SMAS) and development and validation of a shorter scale (SMAS-S) among older patients shortly after hospitalisation. Health Qual Life Outcomes 2012;10. https://doi.org/10.1186/1477-7525-10-9.

[24] Schuurmans H, Steverink N, Frieswijk N, Buunk BP, Slaets JPJ, Lindenberg S. How to measure self-management abilities in older people by self-report. The development of the SMAS-30. Qual Life Res 2005;14:2215–28. https://doi.org/10.1007/s11136-005-8166-9.

[25] Veer IM, Riepenhausen A, Zerban M, Wackerhagen C, Puhlmann LMC, Engen H, et al. Psycho-social factors associated with mental resilience in the Corona lockdown. Transl Psychiatry 2021;11:1–11. https://doi.org/10.1038/s41398-020-01150-4.

[26] Carver CS. You want to measure coping but your protocol's too long: consider the brief COPE. Int J Behav Med 1997;4:92–100. https://doi.org/10.1207/s15327558ijbm0401_6.

[27] Garnefski N, Kraaij V. Cognitive emotion regulation questionnaire - development of a short 18-item version (CERQ-short). Pers Individ Dif 2006;41:1045–53. https://doi.org/10.1016/j.paid.2006.04.010.

[28] Papoula P-R, Engen H, Rupanova A, Tuescher O, Basten U, Kalisch R. Development and validation of self-report assessment of positive appraisal style, 2021.

[29] Turner ML, Engle RW. Is working memory capacity task dependent? J Mem Lang 1989;28:127–54. https://doi.org/10.1016/0749-596X(89)90040-5.

[30] Oswald FL, McAbee ST, Redick TS, Hambrick DZ. The development of a short domain-general measure of working memory capacity. Behav Res Methods 2015;47:1343–55. https://doi.org/10.3758/s13428-014-0543-2.

[31] Conway ARA, Kane MJ, Bunting MF, Hambrick DZ, Wilhelm O, Engle RW. Working memory span tasks: A methodological review and user's guide. Psychon Bull Rev 2005;12:769–86. https://doi.org/10.3758/BF03196772.

[32] Ajzen I. The theory of planned behavior. Organ Behav Hum Decis Process 1991;50:179–211. https://doi.org/10.1016/0749-5978(91)90020-T.

[33] Deci EL, Ryan RM. Hedonia, eudaimonia, and well-being: An introduction. J Happiness Stud 2008;9.

[34] Wilhelm P, Schoebi D. Assessing mood in daily life: Structural validity, sensitivity to change, and reliability of a short-scale to measure three basic dimensions of mood. Eur J Psychol Assess 2007;23:258–67. https://doi.org/10.1027/1015-5759.23.4.258.

[35] Steyer, R., Schwenkmezger, P., Notz, P., & Eid M. metheval - MDMQ questionnaire (English version of MDBF). Https://WwwMethevalUni-Jena.de/MdbfPhp n.d.

https://www.metheval.uni-jena.de/mdbf.php (accessed December 7, 2021).

[36] Brewin CR, Bloomfield MAP, Billings J, Harju-Seppänen J, Greene T. What symptoms best predict severe distress in an online survey of UK health and social care staff facing COVID-19: Development of the two-item Tipping Point Index. BMJ Open 2021;11:e047345. https://doi.org/10.1136/bmjopen-2020-047345.

[37] Gardner B, Abraham C, Lally P, de Bruijn G-J. Towards parsimony in habit measurement: Testing the convergent and predictive validity of an automaticity subscale of the Self-Report Habit Index. Int J Behav Nutr Phys Act 2012;9:1–12. https://doi.org/10.1186/1479-5868-9-102.
